# Performance and Limitation of Machine Learning Algorithms for Diabetic Retinopathy Screening: Meta-analysis

**DOI:** 10.2196/23863

**Published:** 2021-07-05

**Authors:** Jo-Hsuan Wu, T Y Alvin Liu, Wan-Ting Hsu, Jennifer Hui-Chun Ho, Chien-Chang Lee

**Affiliations:** 1 Shiley Eye Institute and Viterbi Family Department of Ophthalmology University of California San Diego La Jolla, CA United States; 2 Retina Division Wilmer Eye Institute The Johns Hopkins Medicine Baltimore, MD United States; 3 Harvard TH Chan School of Public Health Boston, MA United States; 4 National Yang-Ming University Taipei City Taiwan; 5 Health Data Science Research Group, National Taiwan University Hospital Taipei Taiwan; 6 The Centre for Intelligent Healthcare, National Taiwan University Hospital Taipei Taiwan; 7 Department of Emergency Medicine, National Taiwan University Hospital Taipei Taiwan

**Keywords:** machine learning, diabetic retinopathy, diabetes, deep learning, neural network, diagnostic accuracy

## Abstract

**Background:**

Diabetic retinopathy (DR), whose standard diagnosis is performed by human experts, has high prevalence and requires a more efficient screening method. Although machine learning (ML)–based automated DR diagnosis has gained attention due to recent approval of IDx-DR, performance of this tool has not been examined systematically, and the best ML technique for use in a real-world setting has not been discussed.

**Objective:**

The aim of this study was to systematically examine the overall diagnostic accuracy of ML in diagnosing DR of different categories based on color fundus photographs and to determine the state-of-the-art ML approach.

**Methods:**

Published studies in PubMed and EMBASE were searched from inception to June 2020. Studies were screened for relevant outcomes, publication types, and data sufficiency, and a total of 60 out of 2128 (2.82%) studies were retrieved after study selection. Extraction of data was performed by 2 authors according to PRISMA (Preferred Reporting Items for Systematic Reviews and Meta-Analyses), and the quality assessment was performed according to the Quality Assessment of Diagnostic Accuracy Studies 2 (QUADAS-2). Meta-analysis of diagnostic accuracy was pooled using a bivariate random effects model. The main outcomes included diagnostic accuracy, sensitivity, and specificity of ML in diagnosing DR based on color fundus photographs, as well as the performances of different major types of ML algorithms.

**Results:**

The primary meta-analysis included 60 color fundus photograph studies (445,175 interpretations). Overall, ML demonstrated high accuracy in diagnosing DR of various categories, with a pooled area under the receiver operating characteristic (AUROC) ranging from 0.97 (95% CI 0.96-0.99) to 0.99 (95% CI 0.98-1.00). The performance of ML in detecting more-than-mild DR was robust (sensitivity 0.95; AUROC 0.97), and by subgroup analyses, we observed that robust performance of ML was not limited to benchmark data sets (sensitivity 0.92; AUROC 0.96) but could be generalized to images collected in clinical practice (sensitivity 0.97; AUROC 0.97). Neural network was the most widely used method, and the subgroup analysis revealed a pooled AUROC of 0.98 (95% CI 0.96-0.99) for studies that used neural networks to diagnose more-than-mild DR.

**Conclusions:**

This meta-analysis demonstrated high diagnostic accuracy of ML algorithms in detecting DR on color fundus photographs, suggesting that state-of-the-art, ML-based DR screening algorithms are likely ready for clinical applications. However, a significant portion of the earlier published studies had methodology flaws, such as the lack of external validation and presence of spectrum bias. The results of these studies should be interpreted with caution.

## Introduction

Diabetic retinopathy (DR) is the leading cause of vision impairment and blindness among working-aged people in the world [1]. Approximately one-third of people with diabetes mellitus have signs of DR, among whom one-third have vision-threatening DR (VTDR). A meta-analysis estimated global prevalence of any DR and proliferative diabetic retinopathy (PDR) among patients with diabetes to be 35.4% and 7.5%, respectively [2]. The number of patients with DR is approximately 93 million and is expected to rise to 191 million by 2030, as type 2 diabetes has attained the status of a global pandemic, spreading from affluent industrialized nations to the developing world [3].

Vision impairment due to DR can be significantly reduced if diagnosed in early stages and treated appropriately [4]. However, fewer than 60% of patients with diabetes undergo regular eye examinations at intervals recommended by guidelines due to the high cost and low accessibility of ophthalmologic services [3]. The number of people with diabetes that need regular eye examinations has quadrupled in the past three decades. Therefore, the development of an automatic, low-cost, accurate eye screening tool has become an important public health issue [5]. The gold standard for DR screening is based on clinical examinations by human clinicians or the analysis of color fundus photographs via telemedicine [6]. However, both approaches are time-consuming, labor-intensive, and prone to inconsistency due to inherent human subjectivity [7]. Automated systems that are capable of interpreting color fundus photographs with high sensitivity and specificity are critical for widespread implementation of DR screening, and the rise of artificial intelligence (AI), specifically machine learning (ML), has made such automated approaches a possibility.

ML uses existing data to train a computer to recognize a specific pattern or predict a specific outcome in a new data set [6]. Exploration of automated image analysis can be dated back to 1980, when classical ML methods, such as support vector machines and random forests, were used to detect predefined features [8]. These early ML techniques for detecting DR employed mathematical image transformation techniques and image engineering guided by expert-designed rules [9]. The accuracy of this type of analysis did not reach the standard of clinical application. In recent years, the advent of deep learning (DL), a subtype of ML, has transformed the field of automated image analysis [10]. Briefly, DL methods are representation learning methods that use multilayered neural networks, the performance of which can be enhanced by reiteratively changing the internal parameters [11,12]. Unlike other ML approaches, DL does not require image engineering. It develops its own representations needed for pattern recognition after being fed raw data and has shown superior accuracy as compared with other classical ML algorithms [13,14].

Although ML has garnered significant attention with the recent US Food and Drug Administration (FDA) approval of the first ML-based, fully automatic DR screening machine in April 2018 [15], skepticism within the medical community remains regarding the robustness of ML techniques in real-world clinical applications. Given that ophthalmology is among the medical disciplines that have reaped the most benefits from recent AI advancements and that DR screening is one of the most promising ML applications in ophthalmology, we have set out to systematically survey, through meta-analysis, the current status of ML as applied in DR screening based on color fundus photographs. Specifically, we have examined the range of performances reported by different studies and have determined which ML technique is superior for this clinical purpose.

## Methods

### Search Methods for Identifying Studies

This meta-analysis was performed in accordance with the PRISMA (Preferred Reporting Items for Systematic Reviews and Meta-Analyses) guidelines [16]. A literature search for relevant studies published through June 2020 was performed with 2 publicly available databases, PubMed and EMBASE. There were 3 stages to the literature search. No language or population filters were applied, while nonhuman experiments, case reports, guidelines, conference papers, letters, editorials, and review articles were excluded. Filter for publication year was applied only in the second and third stages of the literature search in order to avoid overlapping of search results. Duplicated references in different stages of the literature search were manually excluded. The major search key combination terms were “diabetic retinopathy” OR “diabetic macular edema” OR “macular edema” OR “retinopathy” OR “neovascularized retinopathy” OR “proliferative retinopathy” OR “referable diabetic retinopathy” OR “diabetic macular oedema” OR “proliferative diabetic retinopathy” OR “retinal disorders” OR “diabetic eye disease” OR “vision loss” OR “retinal diseases” OR “macular disease” OR “macular degeneration” OR “macular disorders” crossed with “artificial intelligence” OR “deep learning” OR “transfer learning” OR “machine learning” OR “deep learning system”. The detailed search strategy is provided in Multimedia Appendix 1.

### Eligibility Criteria for Considering Studies for This Review

We included studies that evaluated ML algorithms on the accuracy of automated image analysis for screening or diagnosis of DR. We included studies that detected pathological findings of DR, diagnosed DR status, and staged DR severity.

### Study Selection

The study selection and data extraction were independently performed by 2 authors (JHW and CCL). After duplicates were removed, titles and abstracts were screened for exclusion of studies with potentially nonrelevant outcome or publication types and studies applying information other than images in analytical work. When there were multiples studies derived from the same cohort with overlapping study periods, earlier studies were considered duplicates and only the study with the most recent result was included. Retrieved studies with accessible full articles then underwent full-text review. Discrepancies between the reviewers were resolved first by a consensus meeting and then arbitration by a third reviewer if consensus could not be reached.

### Data Collection

Data extraction was performed on studies selected after full-text review. A thorough review of each article was performed with the following variables extracted: first author, published year, country, algorithm, image modality, total image size, relevant image size, number of participants and eyes, number of diseased participants and eyes, databases and characteristics, and the sensitivity and specificity of both training and validation sessions. When multiple algorithms were tested on one data set, only the data of the best-performing algorithm were included. Algorithms applied were further classified into 4 main categories: support vector machine (SVM), neural network (NN), random forest (RF), and others. After data extraction, studies were classified into different outcomes of DR, including any DR, more-than-mild diabetic retinopathy (mtmDR), vision-threatening diabetic retinopathy (VTDR), diabetic macular edema, and proliferative diabetic retinopathy (PDR). Studies with relevant data were examined for sufficiency to construct a 2 × 2 contingency table before quality assessment.

### Risk of Bias Assessment

The quality of eligible studies was independently assessed by 2 reviewers using the Quality Assessment of Diagnostic Accuracy Studies 2 (QUADAS-2) tool, which is composed of 4 domains assessing both risk of bias and applicability of clinical practice: patient selection, index test, reference standard, and flow and timing. For each diagnostic study, we determined the risk for bias and general applicability in all 4 domains of QUADAS-2 and reported them separately. A study was considered to have a low risk of bias in one domain if at least half of the variables extracted from the validation session met the requirements of QUADAS-2.

### Data Synthesis and Analysis

Meta-analysis for diagnostic accuracy of each ML algorithm or DR outcome was performed with a bivariate random effects model to account for both within- and between-study heterogeneity. Results were summarized by using hierarchical summary receiver operating characteristic (ROC) plots and coupled forest plots. Pooled sensitivity, specificity, area under the curve, and positive and negative likelihood ratios were calculated. The bivariate model approach modeled the logit-transformed sensitivity and specificity simultaneously to account for the inherent negative correlation between sensitivity and specificity that might have arisen due to different thresholds in different studies. Heterogeneity was tested using the Cochran *Q* statistic (*P*<.05) and quantified with the *I^2^* statistic, which describes the variation of effect size that is attributable to heterogeneity across studies. For direct clinical interpretation, we also calculated the posttest probability for each type of lesion. We used the prevalence of lesions in the pooled study population as the informative prior, and derived the posttest probability of each type of lesion based on the pooled positive and negative likelihood ratios. Results are presented as Fagan nomograms. The presence and effect of publication bias were examined with Deeks tests. When publication bias is present, Deeks funnel plots are usually asymmetrical. We used a trim-and-fill method to impute hypothetical missing studies due to publication bias. Trim-and-fill odds ratios (ORs) were reported when the tests for publication bias were significant. We performed sensitivity analyses to examine the potential effects of different demographic factors, ML algorithms, and types of training or validating databases. All analyses except for the summary ROC curve were conducted by the “mada” package in R software (The R Foundation for Statistical Computing). Summary ROC and area under the ROC (AUROC) were calculated by the “midas” package in Stata 14.0 (StataCorp). A 2-sided *P* value <0.05 indicated statistical significance for all tests.

## Results

### Search Results

**Figure 1:**
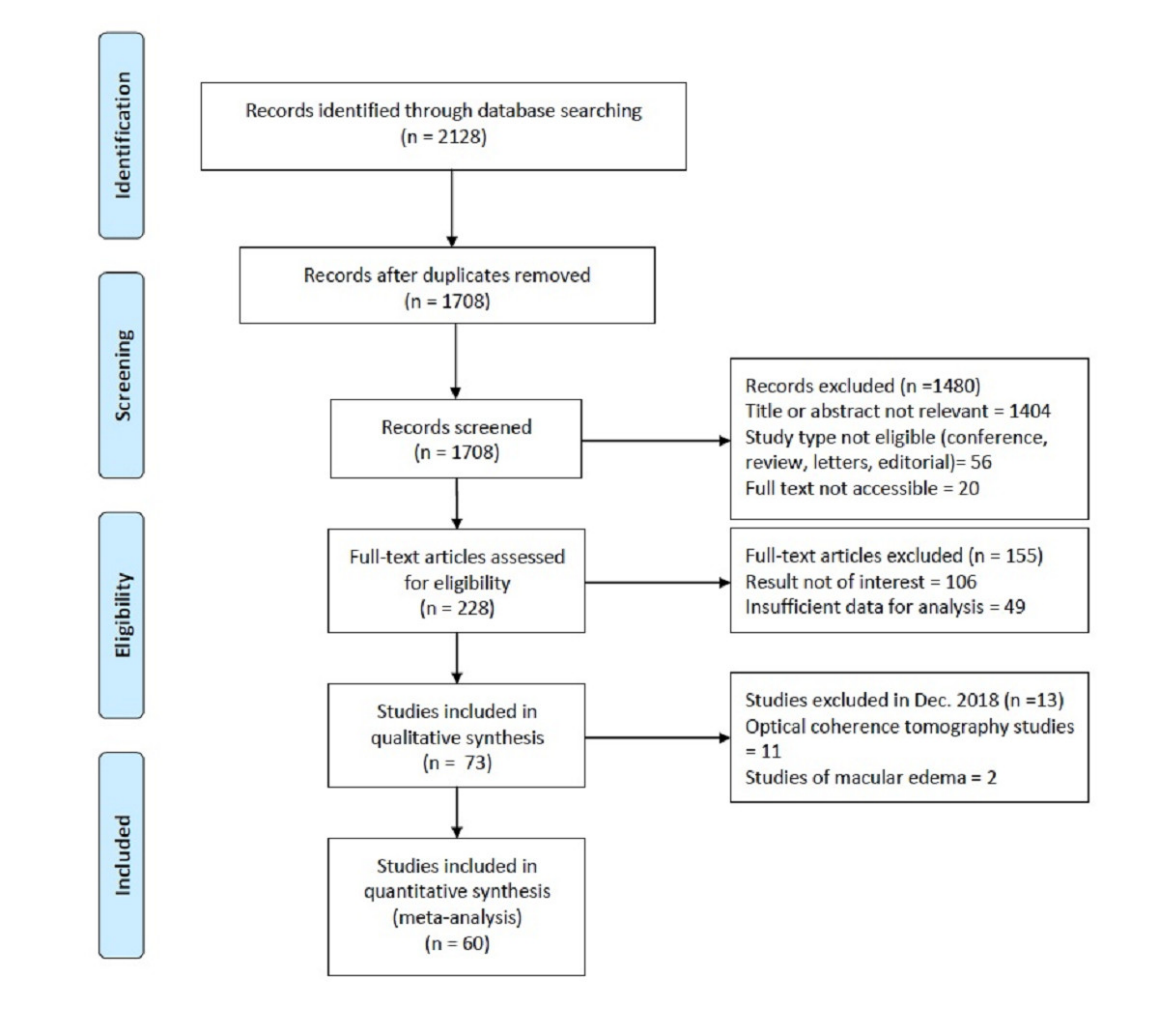
Flowchart of study selection.

The first 2 stages of the literature search (performed in May 2018 and December 2018) yielded 668 hits from PubMed and 809 hits from EMBASE. After screening titles and abstracts, we excluded 336 studies for duplication and 941 studies for nonrelevant abstracts or publication types. A total of 187 studies went through full-text review, 99 of which were excluded for a result that was not of interest and 43 of which were excluded for insufficient data for analysis. After completing qualitative synthesis of the 45 studies, we proceeded to only include ML studies that involved use of color fundus photograph for DR screening. After further exclusion, only 32 studies were retrieved after the second stage of literature selection [13,15,17-46]. The third stage of the literature search was performed in June 2020, and 28 out of 651 studies were retrieved after literature selection [47-74], resulting in a total of 60 (N=32+28) included studies for final analysis. A new category composed of VTDR and PDR (VTDR+PDR) was created for examination of diagnostic accuracy of the most treatment-urgent group. A flowchart of the literature search and study selection process is summarized in Figure 1.

### Study Characteristics

Multimedia Appendix 2 Table S1 summarizes the study-level characteristics of studies assessing the diagnostic accuracy of ML algorithms for different categories of DR. Of the 60 studies, 35 studies (58%) evaluated any DR, 23 (38%) mtmDR, 12 (20%) VTDR, and 12 (20%) PDR. Publicly available benchmark databases, such as Messidor, Structured Analysis of the Retina (STARE), Digital Retinal Images for Vessel Extraction (DRIVE), DIARETDB, e-Ophtha, and EyePACs were used for testing of the ML algorithms in 40 of the 60 (67%) studies. The characteristics of these publicly available retinal image databases are summarized in Multimedia Appendix 3. The distribution of categories of ML algorithms used was as follows: SVM (6/60, 10%), RF (2/60, 3%), NN (37/60, 62%), and others (17/60, 28%). The general principles of these ML algorithms are described in Multimedia Appendix 4.

### Quality Assessment

Quality assessments using the QUADAS-2 criteria are summarized in Multimedia Appendix 5. Most studies (56/60, 93%) presented a clear source of patient recruitment or selection criteria and processes, and were at a low risk for bias. Of the 60 studies, 3 (5%) reported limited information on the establishment of reference standard and were at a high risk for bias, and 4 (7%) reported insufficient blinding to a reference standard during interpretation of the index test results and were at a high risk for bias. For study applicability, 1 study (2%) in the index test section and 4 (7%) studies in the patient selection section were recorded to be at a high risk of concern, due to insufficient information reported.

### Synthesis of Results

**Figure 2:**
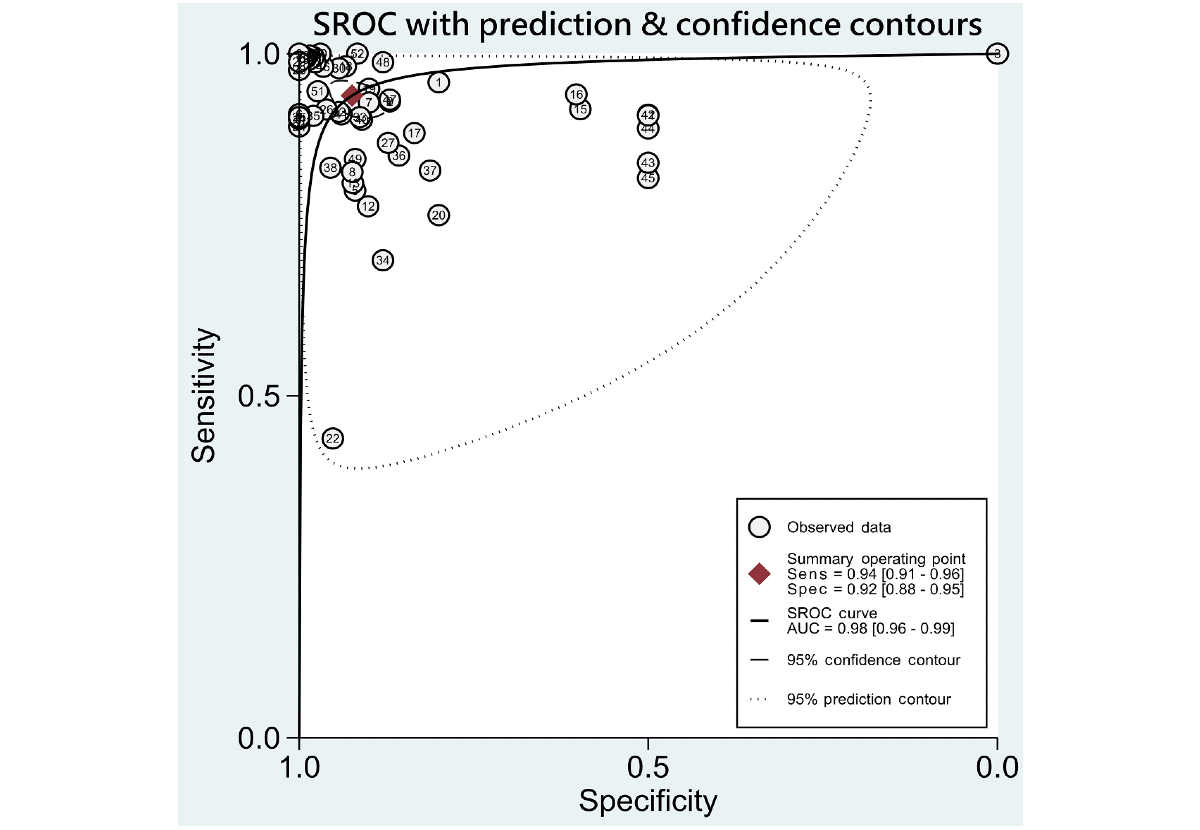
SROC curves for diagnosis of any diabetic retinopathy on color fundus photographs. AUC: area under the curve; Sens: sensitivity; Spec: specificity; SROC: summary receiver operating characteristics.

**Figure 3:**
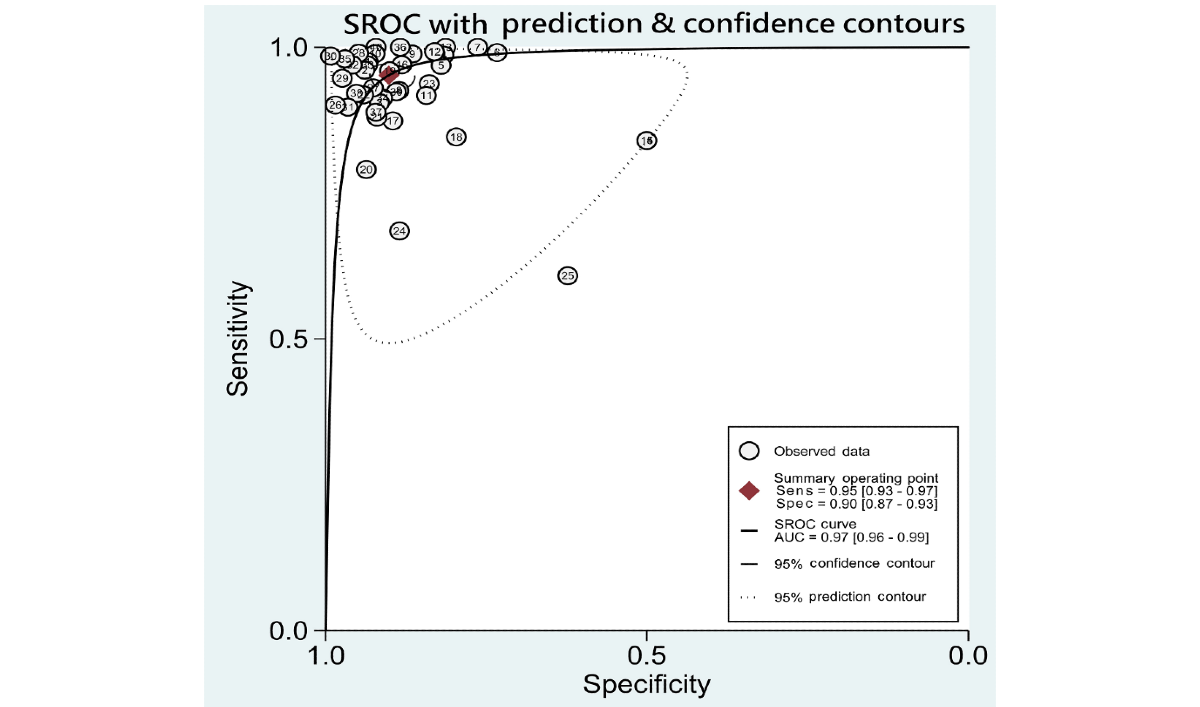
SROC curves for diagnosis of more-than-mild diabetic retinopathy on color fundus photographs. Sens: sensitivity; Spec: specificity; SROC: summary receiver operating characteristics; AUC: area under the curve.

**Figure 4:**
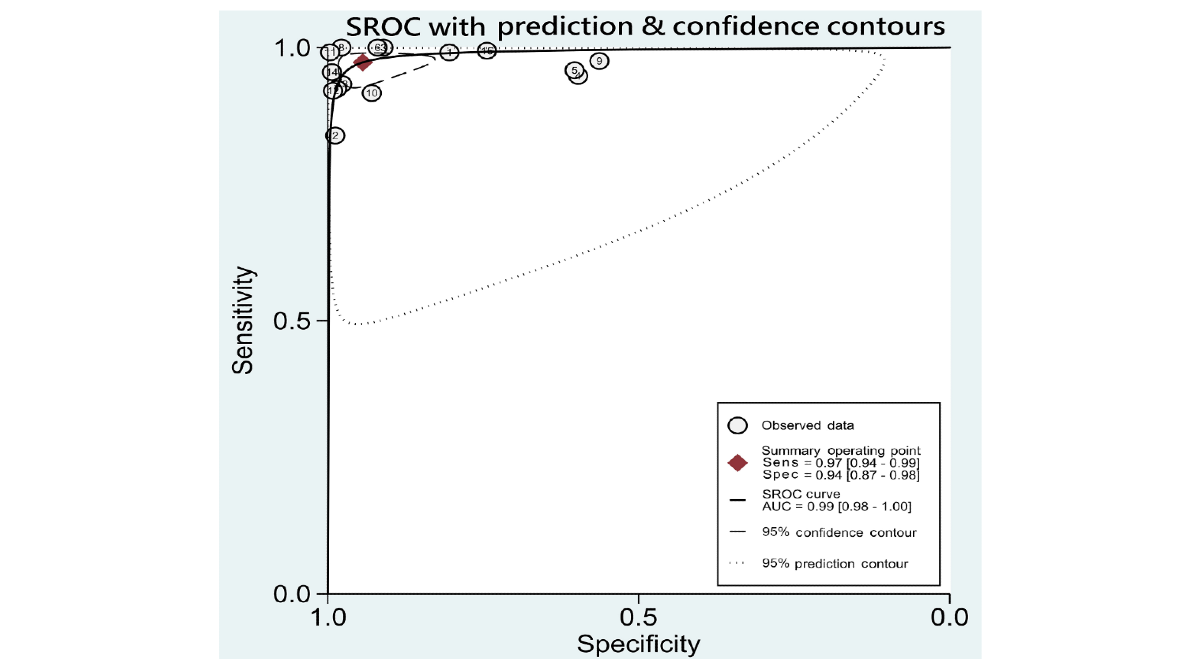
SROC curves for diagnosis of vision-threatening diabetic retinopathy on color fundus photographs. AUC: area under the curve; Sens: sensitivity; Spec: specificity; SROC: summary receiver operating characteristics.

**Figure 5:**
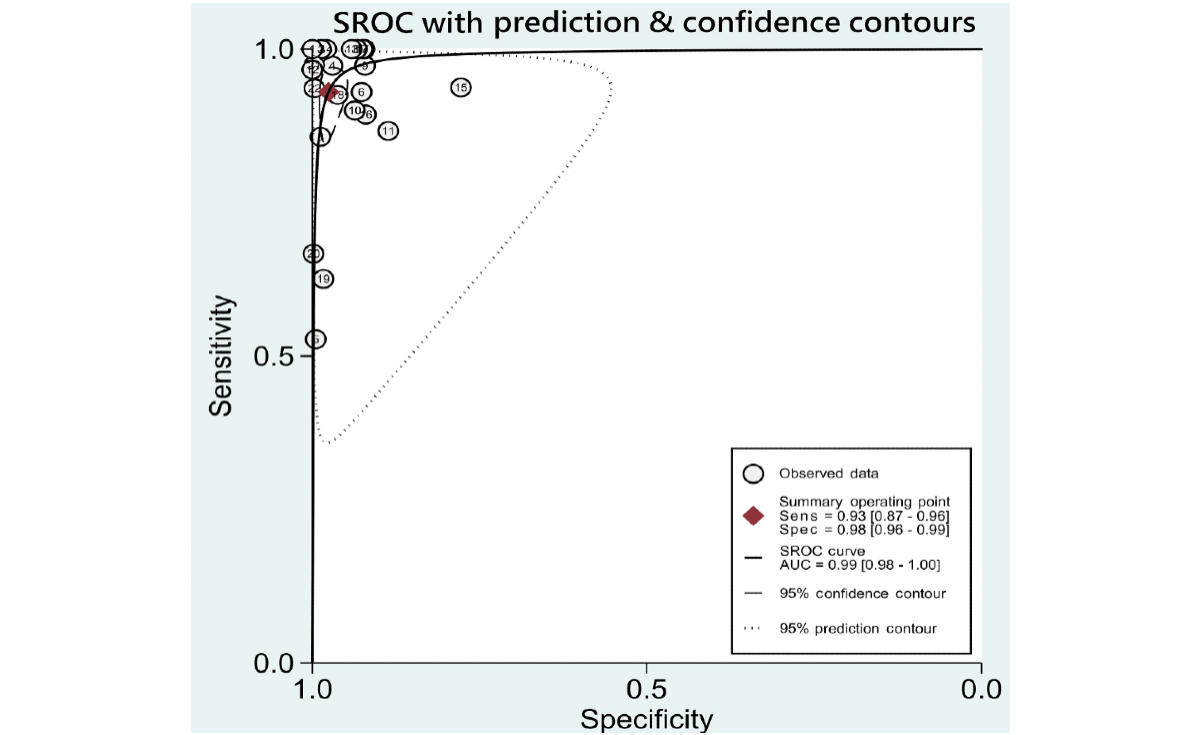
SROC curves for diagnosis of proliferative diabetic retinopathy on color fundus photographs. AUC: area under the curve; Sens: sensitivity; Spec: specificity; SROC: summary receiver operating characteristics.

A summary of data of included studies is presented in Multimedia Appendix 6. Pooled sensitivities, specificities, likelihood ratios, AUROCs, and *I*^2^ statistics for the 5 DR categories, including any DR, mtmDR, VTDR, PDR, and VTDR+PDR, are presented in Table 1. As some studies might have used more than 2 data sets for validation, performance of ML derived from each data set was viewed as individual data, and we used “data” as the unit for calculation (eg, 35 included studies performed evaluation of ML on identifying any DR, resulting in a total of 53 data for synthesis and analysis). The hierarchical summary ROC plots for the 4 main DR categories, including DR, mtmDR, VTDR, and PDR, are also presented (Figures 2-5). ML showed a high overall accuracy in detecting the 5 categories of DR, with a pooled AUROC ranging from 0.97 (95% CI 0.96-0.99) for mtmDR and VTDR+PDR to 0.99 (95% CI 0.98-1.00) for VTDR and PDR. The pooled sensitivity for all 5 categories was high, ranging from 0.93 (95% CI 0.87-0.96) for PDR to 0.97 (95% CI 0.94-0.99) for VTDR. The pooled specificity, however, showed more variation: from 0.90 (95% CI 0.87-0.93) for mtmDR to 0.98 (95% CI 0.96-0.99) for PDR. The Fagan plots for different DR categories are presented in Multimedia Appendix 7. For images that were classified as positive by the ML algorithms, the posttest probability for DR, mtmDR, VTDR, and PDR was 87%, 71%, 66%, and 77%, respectively. For images that were classified as negative by the ML algorithms, the posttest probability for DR, mtmDR, VTDR, and PDR was 4%, 1%, 0%, and 1%, respectively.

**Table 1:** Pooled analysis for diagnostic accuracy of diabetic retinopathy by machine learning on color fundus photographs.

Goal of detection	Data^a^, n	Sen^b,c^	Spe^d,c^	LR+^e,c^	LR–^f,c^	AUROC^g,c^	*I*^2^ statistic^c^	Publication bias (*P* value)
Any DR^h^	53	0.94 (0.91-0.96)	0.92 (0.88-0.95)	12.4 (8.0-19.3)	0.07 (0.05-0.09)	0.98 (0.96-0.99)	32 (22-42)	.01
mtmDR^i^	40	0.95 (0.93-0.97)	0.90 (0.87-0.93)	9.7 (7.4-12.7)	0.05 (0.04-0.08)	0.97 (0.96-0.99)	29 (18-40)	.11
VTDR^j^	15	0.97 (0.94-0.99)	0.94 (0.87-0.98)	17.3 (7.5-39.9)	0.03 (0.01-0.06)	0.99 (0.98-1.00)	32 (9-56)	.33
PDR^k^	22	0.93 (0.87-0.96)	0.98 (0.96-0.99)	38.5 (21.7-68.4)	0.07 (0.04-0.13)	0.99 (0.98-1.00)	29 (11-46)	.11
VTDR and PDR	37	0.96 (0.93-0.98)	0.97 (0.94-0.98)	24.3 (14.5-38.5)	0.07 (0.05-0.10)	0.97 (0.96-0.99)	N/A	.06

^a^Machine learning data derived from each data set was viewed as individual data, and we used “data” as the unit for calculation.

^b^Sen: sensitivity.

^c^Values in this column are as follows: mean (95% confidence interval).

^d^Spe: specificity.

^e^LR+: positive likelihood ratio.

^f^LR–: negative likelihood ratio.

^g^AUROC: area under the receiver operating characteristic.

^h^DR: diabetic retinopathy.

^i^mtmDR: more-than-mild diabetic retinopathy.

^j^VTDR: vision-threatening diabetic retinopathy.

^k^PDR: proliferative diabetic retinopathy.

### Subgroup Analyses and Sensitivity Analysis

We performed subgroup analyses for mtmDR studies to explore the possible heterogeneity in test accuracy (Table 2). The main causes of heterogeneity included in the analysis were algorithm type, mean age of subject populations, and validation set selection. For this subgroup analysis, 23 studies were included, with a total of 40 data obtained from different testing data sets. Of the 23 studies, the 22 studies that applied NN algorithms demonstrated high pooled performance (summary AUROC 0.98; 95% CI 0.96-0.99), sensitivity (sensitivity 0.95; 95% CI 0.93-0.97), and specificity (specificity 0.91; 95% CI 0.88-0.93). The only study that used a different kind of ML algorithm (instance learning) reported significantly inferior sensitivity (0.84) under preset specificity (0.50). Of the 60 studies, 19 (83%) tested the algorithm’s performance on data sets with subject populations with a mean age greater than 50 years. Pooled sensitivity of data from these studies was high (sensitivity 0.95; 95% CI 0.93-0.97), and the pooled specificity was moderate (specificity 0.89; 95% CI 0.85-0.92). Compared with algorithms that used benchmark data sets for validation (pooled sensitivity 0.92; 95% CI 0.87-0.95), the pooled sensitivities of algorithms validated by clinical data sets (sensitivity 0.97; 95% CI 0.95-0.98) and independent data sets (sensitivity 0.96; 95% CI 0.93-0.97) were not inferior. The results of pooled AUROCs validated by these 3 types of data sets were similar, implying that robust performance of ML algorithms can be generalized to images collected in clinical practice.

**Table 2:** Subgroup analysis for diagnostic accuracy of mtmDR retinopathy on color fundus photographs.

Features of subgroup	Data^a^, n	Sen^b,c^	Spe^d,c^	LR+^e,c^	LR–^f,c^	AUROC^g,c^	*I*^2^ statistic^c^	Publication bias (*P* value)
Overall mtmDR^h^	40	0.95 (0.93, 0.97)	0.90 (0.87, 0.93)	9.7 (7.4, 12.7)	0.05 (0.04, 0.08)	0.97 (0.96, 0.99)	29 (18, 40)	.11
Mean age> 50 years	32	0.95 (0.93, 0.97)	0.89 (0.85, 0.92)	8.8 (6.4, 12.0)	0.05 (0.03, 0.08)	0.97 (0.95, 0.98)	33 (20, 46)	.22
NN^i^ algorithms	38	0.95 (0.93, 0.97)	0.91 (0.88, 0.93)	10.1 (7.7, 13.2)	0.05 (0.03, 0.07)	0.98 (0.96, 0.99)	30 (19, 41)	.14
Benchmark test sets	15	0.92 (0.87, 0.95)	0.90 (0.82, 0.94)	9.0 (4.8, 16.6)	0.09 (0.05, 0.16)	0.96 (0.94, 0.98)	25 (10, 39)	.22
Clinical Test sets	25	0.97 (0.95, 0.98)	0.90 (0.88, 0.92)	10.0 (7.9, 12.6)	0.04 (0.02, 0.06)	0.97 (0.96, 0.98)	30 (15, 45)	.06
External validation	31	0.96 (0.93, 0.97)	0.90 (0.87, 0.93)	9.7 (7.2, 13.0)	0.05 (0.03, 0.07)	0.98 (0.96, 0.99)	29 (16, 42)	.08

^a^Machine learning data derived from each data set was viewed as individual data, and we used “data” as the unit for calculation.

^b^Sen: sensitivity.

^c^Values in this column are as follows: mean (95% confidence interval).

^d^Spe: specificity.

^e^LR+: positive likelihood ratio.

^f^LR–: negative likelihood ratio.

^g^AUROC: area under the receiver operating characteristic.

^h^mtmDR: more-than-mild diabetic retinopathy.

^i^NN: neural network.

### Publication Bias

The test for publication bias was generally not significant in different categories of DR (Deeks test *P*=.01; Multimedia Appendix 8), except for any DR. Trim-and-fill analysis showed the diagnostic OR remained insignificant (OR 0.50, 95% CI 0.25-1.01) after hypothetical unpublished data were included for analysis (Multimedia Appendix 9).

## Discussion

### Principal Results and Comparison With Prior Work

This systematic review synthesizes the available evidence and compares the diagnostic accuracy of ML algorithms for the detection of DR based on color fundus photographs. The primary meta-analysis included 60 studies with 445,175 interpretations. Out of the 60 studies, 35 (58%) were validated by external testing data sets that were completely independent of the training data sets. Overall, ML demonstrated a robust performance in detecting different DR categories, with a pooled sensitivity of 0.93 to 0.97 and a pooled specificity of 0.90 to 0.98. The pooled sensitivity compares favorably to reported sensitivities of 73% [75], 34% [76], and 33% [77] achieved by board-certified ophthalmologists performing indirect ophthalmoscopy and to reported sensitivities of 92% [78] and 89% [79] achieved by ophthalmologists interpreting digital fundus photographs. Our analysis suggests that the performance of ML algorithms in detecting DR based on color fundus photographs is likely to be on par with human clinicians and supports a previous study that compared humans head to head with ML. Rajalakshmi et al [37] compared the performance of an AI DR screening software (EyeArt) on smartphone-based fundus photographs of 296 patients to the performance of human graders who evaluated the same data set. The EyeArt achieved a high sensitivity of 95.8% for any retinopathy and 99.1% for VTDR, both of which were on par with human graders. Our pooled data suggest that ML techniques are more sensitive than specific in DR detection. It is unclear whether this is a reflection of the limitations of ML techniques for this clinical purpose or whether it is by design. It is possible that model developers of these studies chose optimal statistical thresholds that favored sensitivity over specificity. Regardless, the lower specificity should not pose a major issue, as false negatives are much more problematic than are false positives in the context of disease screening. Furthermore, the major causes of false positives in retinal image interpretation, including inadequate image quality and artifacts [55], are modifiable with future improvement in image quality control.

To further facilitate direct clinical interpretations, we used Fagan nomograms to determine whether a patient with a positive or negative finding by ML actually has that particular finding as per the gold standard. For any DR, in a population with a DR prevalence of 36%, a positive likelihood ratio of 12.4 translates into a posttest probability of 87%. In other words, approximately 9 out of 10 patients with a positive ML diagnosis of DR can be expected to have DR as per the gold standard. The diagnostic value for ML to rule out DR performed as well as its rule-in value. In the same population, a negative ML diagnosis translates into a 4% posttest probability of any DR (negative likelihood ratio 0.07) and only a 1% posttest probability of mtmDR (negative likelihood ratio 0.05). These numbers again suggest that ML is extremely sensitive in detecting overall DR and mtmDR based on color fundus photographs and that the rate of false negatives are low.

We performed an in-depth analysis of studies that involved the detection of mtmDR, as Abramoff et al’s [15] pivotal trial involved the detection of mtmDR and led to the FDA’s approval of the first fully automated ML system. Among the 16 mtmDR studies conducted by other research teams that were also externally validated, 14 showed performance superior to the preset end points (sensitivity >85%; specificity > 82.5%) used in Abramoff et al’s trial. Although only 5 out of these studies were prospectively evaluated in a real-world setting as the Abramoff algorithm was, this suggests that Abramoff et al’s trial was no accident and that ML algorithms in general are likely capable of producing clinical grade detections of mtmDR based on color fundus photographs. In addition, no statistically significant difference in pooled AUROC between studies validated by benchmark databases and studies validated by clinical databases was identified within this group.

To the best of our knowledge, previous meta-reviews on DR screening have focused on the performance of DL algorithms alone [80,81]. DL is only a subtype of ML, and other ML techniques, such as SVM and RF, can be used to detect DR as well. Therefore, our meta-analysis was more comprehensive than these previous studies, as it included all ML studies, including DL studies, published through 2020. In addition, the review by Nielsen et al [80] did not conduct pooled analysis on the results of past studies, while our study did. The meta-analysis by Islam et al [81] focused mainly on detection of referable DR, while our study was broader and more fine grained, as it evaluated the ability of ML to detect different categories of DR, including any DR, mtmDR (referrable DR), VTDR, and PDR. These analyses are clinically meaningful, as different categories of DR require different management strategies. For example, while patients with moderate nonproliferative DR (a subset within referrable DR) should be further evaluated by ophthalmologists at some point, patients with VTDR require immediate referrals to retinal specialists.

### Use and Predominance of NN Algorithms

NN algorithms, especially deep convolutional NN algorithms, were generally recognized as the best ML technique for automated medical image analysis. NN algorithms were also the most-used technique in diagnosing DR of all categories in our study, being used in 37 of the 60 (62%) studies. As for the 23 studies evaluating mtmDR (Table 2), NN algorithms were used in 22 studies and contributed to the high pooled AUROC of 0.98 (95% CI 0.96-0.99). In addition, we ranked the performance of the included studies by sensitivity, specificity, and quality. The top-5 performing, high-quality (based on QUADUS-2 and study design) studies are listed in Multimedia Appendix 10, and 4 out of the 5 studies used NN algorithms. This result confirms that NN is the cutting-edge ML technique for medical image classification, at least in the context of DR detection.

### Limitations

Our study was based on a rigorous literature search, and a validated appraisal tool was used to determine the risk of bias of included studies. Several limitations should be considered, however. First, of the 60 studies included for final analysis, only 35 applied true external validation. For those studies without external validation, the generalizability of their ML algorithms was not adequately evaluated, and thus their reported performance should be interpreted with caution. Second, without sufficient details, we were unable to conduct subgroup analysis on populations with available key factors of DR that could influence the clinical practicability of the diagnostic tool. Bias could have been introduced by poor reporting of patient characteristics of the included studies. Finally, except for Abramoff et al’s trial [15] and 5 other prospectively conducted studies [48,52,55,60,66], all other studies on ML-based DR diagnosis were validated by retrospective data. Due to spectrum bias, an overestimation of ML’s performance in a real-world setting is possible and should be considered.

### Conclusions

ML algorithms for diagnosing DR based on color fundus photographs have shown high diagnostic accuracy for different categories of DR. Specifically, the performances of ML algorithms in detecting mtmDR, the widely accepted threshold for clinically relevant DR, compare favorably to those of clinical examinations by ophthalmologists and to those of expert grading of digital fundus photographs. To the best of our knowledge, this is the first meta-analysis in the published literature that quantitatively assessed the performance of ML algorithms for a specific medical image classification task. As evidence-based medicine expands from therapy to diagnosis, the information from this systematic review may provide important evidence in the determination of the proper and efficacious use of ML algorithms in the diagnosis or screening of DR and may serve as a framework for similar analyses of other medical conditions conducted in the future. However, our meta-analysis also showed that a significant portion of the published studies had methodological flaws, such as the lack of external validation and presence of spectrum bias. Therefore, more rigorous prospective studies would be helpful in establishing the true efficacy of these algorithms in real-life clinical care.

## Appendix

Multimedia Appendix 1 Search detail for PubMed and EMBASE.

Multimedia Appendix 2 Summary of characteristics of the included studies.

Multimedia Appendix 3 Characteristics of public databases for benchmarking diabetic retinopathy detection from color fundus photographs.

Multimedia Appendix 4 Principles of major machine learning algorithms and methods applied in automated image analysis.

Multimedia Appendix 5 Summary of QUADAS-2 assessment for included studies.

Multimedia Appendix 6 Summary of data of included studies.

Multimedia Appendix 7 Fagan plot for diagnosis of different categories of diabetic retinopathy on color fundus photographs.

Multimedia Appendix 8 Deeks funnel plot for 4 main types of diabetic retinopathy lesions on color fundus photograph.

Multimedia Appendix 9 A filled funnel plot that filled the potential missing studies (square) due to publication bias.

Multimedia Appendix 10 Characteristics and best results of 5 high-quality studies with top performances.

## Abbreviations

AI: artificial intelligence
AUROC: area under the receiver operating characteristics
DL: deep learning
DR: diabetic retinopathy
DRIVE: Digital Retinal Images for Vessel Extraction
FDA: US Food and Drug Administration
ML: machine learning
mtmDR: more-than-mild diabetic retinopathy
NN: neural network
OR: odds ratio
PDR: proliferative diabetic retinopathy
PRISMA: Preferred Reporting Items for Systematic Reviews and Meta-Analyses
QUADAS-2: Quality Assessment of Diagnostic Accuracy Studies 2
RF: random forest
ROC: receiver operating characteristics
STARE: Structured Analysis of the Retina
SVM: support vector machine
VTDR: vision-threatening diabetic retinopathy
