# Supplementary material for: Performance and Limitation of Machine Learning Algorithms for Diabetic Retinopathy Screening: Meta-analysis
Source: J Med Internet Res. 2021 Jul 5;23(7):e23863. doi: 10.2196/23863 (PMC8406115; doi:10.2196/23863)
Supplement: Multimedia Appendix 1 [file jmir_v23i7e23863_app1.docx]

Method 1. Search detail of first-stage search for PubMed and EMBASE

1A. Search detail for PubMed

Search (((((((((((((((((((((((((((((((((((((((diabetic retinopathy) OR diabetic macular edema) OR macular edema) OR macular degeneration) OR age-related macular degeneration) OR retinopathy) OR neovascularized retinopathy) OR proliferative retinopathy) OR geographic atrophy) OR drusen) OR choroidal neovascularization) OR referable diabetic retinopathy) OR glaucoma)) OR diabetic macular oedema) OR proliferative diabetic retinopathy) OR retinal disorders) OR diabetic eye disease) OR vision loss) OR retinal diseases)) OR AMD) OR macular disease) OR macular degeneration) OR macular disorders))))) AND (((((((((artificial intelligence) OR deep learning) OR transfer learning) OR machine learning) OR deep learning system)) OR deep convolutional neural network))))) NOT (((((((((((((((((((((((((((((((diabetic retinopathy) OR diabetic macular edema) OR macular edema) OR macular degeneration) OR age-related macular degeneration) OR retinopathy) OR neovascularized retinopathy) OR proliferative retinopathy) OR geographic atrophy) OR drusen) OR choroidal neovascularization) OR referable diabetic retinopathy) OR glaucoma)) OR diabetic macular oedema) OR proliferative diabetic retinopathy) OR retinal disorders) OR diabetic eye disease) OR vision loss) OR retinal diseases)) OR AMD) OR macular disease) OR macular degeneration) OR macular disorders))))) AND (((((((((artificial intelligence) OR deep learning) OR transfer learning) OR machine learning) OR deep learning system)) OR deep convolutional neural network)))) AND Review[ptyp]))) NOT (((((((((((((((((((((((((((((((diabetic retinopathy) OR diabetic macular edema) OR macular edema) OR macular degeneration) OR age-related macular degeneration) OR retinopathy) OR neovascularized retinopathy) OR proliferative retinopathy) OR geographic atrophy) OR drusen) OR choroidal neovascularization) OR referable diabetic retinopathy) OR glaucoma)) OR diabetic macular oedema) OR proliferative diabetic retinopathy) OR retinal disorders) OR diabetic eye disease) OR vision loss) OR retinal diseases)) OR AMD) OR macular disease) OR macular degeneration) OR macular disorders))))) AND (((((((((artificial intelligence) OR deep learning) OR transfer learning) OR machine learning) OR deep learning system)) OR deep convolutional neural network)))) AND systematic[sb]))) NOT (((((((((((((((((((((((((((((((diabetic retinopathy) OR diabetic macular edema) OR macular edema) OR macular degeneration) OR age-related macular degeneration) OR retinopathy) OR neovascularized retinopathy) OR proliferative retinopathy) OR geographic atrophy) OR drusen) OR choroidal neovascularization) OR referable diabetic retinopathy) OR glaucoma)) OR diabetic macular oedema) OR proliferative diabetic retinopathy) OR retinal disorders) OR diabetic eye disease) OR vision loss) OR retinal diseases)) OR AMD) OR macular disease) OR macular degeneration) OR macular disorders))))) AND (((((((((artificial intelligence) OR deep learning) OR transfer learning) OR machine learning) OR deep learning system)) OR deep convolutional neural network)))) AND Case Reports[ptyp]))) NOT (((((((((((((((((((((((((((((((diabetic retinopathy) OR diabetic macular edema) OR macular edema) OR macular degeneration) OR age-related macular degeneration) OR retinopathy) OR neovascularized retinopathy) OR proliferative retinopathy) OR geographic atrophy) OR drusen) OR choroidal neovascularization) OR referable diabetic retinopathy) OR glaucoma)) OR diabetic macular oedema) OR proliferative diabetic retinopathy) OR retinal disorders) OR diabetic eye disease) OR vision loss) OR retinal diseases)) OR AMD) OR macular disease) OR macular degeneration) OR macular disorders))))) AND (((((((((artificial intelligence) OR deep learning) OR transfer learning) OR machine learning) OR deep learning system)) OR deep convolutional neural network)))) AND Comment[sb]))) NOT (((((((((((((((((((((((((((((((diabetic retinopathy) OR diabetic macular edema) OR macular edema) OR macular degeneration) OR age-related macular degeneration) OR retinopathy) OR neovascularized retinopathy) OR proliferative retinopathy) OR geographic atrophy) OR drusen) OR choroidal neovascularization) OR referable diabetic retinopathy) OR glaucoma)) OR diabetic macular oedema) OR proliferative diabetic retinopathy) OR retinal disorders) OR diabetic eye disease) OR vision loss) OR retinal diseases)) OR AMD) OR macular disease) OR macular degeneration) OR macular disorders))))) AND (((((((((artificial intelligence) OR deep learning) OR transfer learning) OR machine learning) OR deep learning system)) OR deep convolutional neural network)))) AND Editorial[ptyp])

1B. Search detail for EMBASE

('diabetic retinopathy'/exp OR 'diabetic retinopathy' OR (('diabetic'/exp OR diabetic) AND ('retinopathy'/exp OR retinopathy)) OR 'diabetic macular edema'/exp OR 'diabetic macular edema' OR (('diabetic'/exp OR diabetic) AND macular AND ('edema'/exp OR edema)) OR 'macular edema'/exp OR 'macular edema' OR (macular AND ('edema'/exp OR edema)) OR 'age-related macular degeneration'/exp OR 'age-related macular degeneration' OR ('age related' AND macular AND ('degeneration'/exp OR degeneration)) OR 'retinopathy'/exp OR retinopathy OR 'neovascularized retinopathy' OR (neovascularized AND ('retinopathy'/exp OR retinopathy)) OR 'proliferative retinopathy'/exp OR 'proliferative retinopathy' OR (proliferative AND ('retinopathy'/exp OR retinopathy)) OR 'geographic atrophy'/exp OR 'geographic atrophy' OR (geographic AND ('atrophy'/exp OR atrophy)) OR 'drusen'/exp OR drusen OR 'choroidal neovascularization'/exp OR 'choroidal neovascularization' OR (choroidaland AND ('neovascularization'/exp OR neovascularization)) OR 'referable diabetic retinopathy' OR (referable AND ('diabetic'/exp OR diabetic) AND ('retinopathy'/exp OR retinopathy)) OR 'glaucoma'/exp OR glaucoma OR 'diabetic macular oedema'/exp OR 'diabetic macular oedema' OR (('diabetic'/exp OR diabetic) AND macular AND ('oedema'/exp OR oedema)) OR 'proliferative diabetic retinopathy'/exp OR 'proliferative diabetic retinopathy' OR (proliferative AND ('diabetic'/exp OR diabetic) AND ('retinopathy'/exp OR retinopathy)) OR 'retinal disorders' OR (('retinal'/exp OR retinal) AND ('disorders'/exp OR disorders)) OR 'diabetic eye disease'/exp OR 'diabetic eye disease' OR (('diabetic'/exp OR diabetic) AND ('eye'/exp OR eye) AND ('disease'/exp OR disease)) OR 'vision loss'/exp OR 'vision loss' OR (('vision'/exp OR vision) AND loss) OR 'retinal diseases'/exp OR 'retinal diseases' OR (('retinal'/exp OR retinal) AND ('diseases'/exp OR diseases)) OR 'amd'/exp OR amd OR 'macular disease'/exp OR 'macular disease' OR (macular AND ('disease'/exp OR disease)) OR 'macular degeneration'/exp OR 'macular degeneration' OR (macular AND ('degeneration'/exp OR degeneration)) OR 'macular disorders'/exp OR 'macular disorders' OR (macular AND ('disorders'/exp OR disorders))) AND ('artificial intelligence'/exp OR 'artificial intelligence' OR (artificial AND ('intelligence'/exp OR intelligence)) OR 'deep learning'/exp OR 'deep learning' OR (deep AND ('learning'/exp OR learning)) OR 'transfer learning'/exp OR 'transfer learning' OR (('transfer'/exp OR transfer) AND ('learning'/exp OR learning)) OR 'machine learning'/exp OR 'machine learning' OR (('machine'/exp OR machine) AND ('learning'/exp OR learning)) OR 'deep learning system' OR (deepand AND ('learning'/exp OR learning) AND system) OR 'deep convolutional neural network'/exp OR 'deep convolutional neural network' OR (deep AND convolutional AND neural AND ('network'/exp OR network))) AND 'human'/de AND ('clinical article'/de OR 'clinical trial'/de OR 'cohort analysis'/de OR 'comparative study'/de OR 'computer simulation'/de OR 'controlled study'/de OR 'diagnostic test accuracy study'/de OR 'human experiment'/de OR 'in vitro study'/de OR 'intermethod comparison'/de OR 'major clinical study'/de OR 'methodology'/de OR 'model'/de OR 'normal human'/de OR 'pilot study'/de OR 'prospective study'/de OR 'randomized controlled trial'/de OR 'retrospective study'/de OR 'validation process'/de OR 'validation study'/de) AND 'article'/it NOT 'nonhuman'/de

Method 2. Search detail of second-stage search in December 2018 for PubMed and EMBASE

2A. Search detail for PubMed

Search (((((diabetic retinopathy OR diabetic macular edema OR macular edema OR retinopathy OR neovascularized retinopathy OR proliferative retinopathy OR referable diabetic retinopathy OR diabetic macular oedema OR proliferative diabetic retinopathy OR retinal disorders OR diabetic eye disease OR vision loss OR retinal diseases OR macular disease OR macular degeneration OR macular disorders) AND (artificial intelligence OR deep learning OR transfer learning OR machine learning OR deep learning system).)) AND ( "2018/05/08"[PDat] : "2018/12/15"[PDat] ) AND Humans[Mesh])) NOT (((((diabetic retinopathy OR diabetic macular edema OR macular edema OR retinopathy OR neovascularized retinopathy OR proliferative retinopathy OR referable diabetic retinopathy OR diabetic macular oedema OR proliferative diabetic retinopathy OR retinal disorders OR diabetic eye disease OR vision loss OR retinal diseases OR macular disease OR macular degeneration OR macular disorders) AND (artificial intelligence OR deep learning OR transfer learning OR machine learning OR deep learning system)))) AND ( ( Case Reports[ptyp] OR Comment[sb] OR Editorial[ptyp] OR Review[ptyp] OR Meta-Analysis[ptyp] OR Letter[ptyp] ) AND full text[sb] AND ( "2018/05/08"[PDat] : "2018/12/15"[PDat] ) AND Humans[Mesh]))

2B. Search detail for EMBASE

('diabetic retinopathy'/exp OR 'diabetic retinopathy' OR (('diabetic'/exp OR diabetic) AND ('retinopathy'/exp OR retinopathy)) OR 'diabetic macular edema'/exp OR 'diabetic macular edema' OR (('diabetic'/exp OR diabetic) AND macular AND ('edema'/exp OR edema)) OR 'macular edema'/exp OR 'macular edema' OR (macular AND ('edema'/exp OR edema)) OR 'retinopathy'/exp OR retinopathy OR 'neovascularized retinopathy' OR (neovascularized AND ('retinopathy'/exp OR retinopathy)) OR 'proliferative retinopathy'/exp OR 'proliferative retinopathy' OR (proliferative AND ('retinopathy'/exp OR retinopathy)) OR 'referable diabetic retinopathy' OR (referable AND ('diabetic'/exp OR diabetic) AND ('retinopathy'/exp OR retinopathy)) OR 'diabetic macular oedema'/exp OR 'diabetic macular oedema' OR (('diabetic'/exp OR diabetic) AND macular AND ('oedema'/exp OR oedema)) OR 'proliferative diabetic retinopathy'/exp OR 'proliferative diabetic retinopathy' OR (proliferative AND ('diabetic'/exp OR diabetic) AND ('retinopathy'/exp OR retinopathy)) OR 'retinal disorders' OR (('retinal'/exp OR retinal) AND ('disorders'/exp OR disorders)) OR 'diabetic eye disease'/exp OR 'diabetic eye disease' OR (('diabetic'/exp OR diabetic) AND ('eye'/exp OR eye) AND ('disease'/exp OR disease)) OR 'vision loss'/exp OR 'vision loss' OR (('vision'/exp OR vision) AND loss) OR 'retinal diseases'/exp OR 'retinal diseases' OR (('retinal'/exp OR retinal) AND ('diseases'/exp OR diseases)) OR 'macular disease'/exp OR 'macular disease' OR (macular AND ('disease'/exp OR disease)) OR 'macular degeneration'/exp OR 'macular degeneration' OR (macular AND ('degeneration'/exp OR degeneration)) OR 'macular disorders'/exp OR 'macular disorders' OR (macular AND ('disorders'/exp OR disorders))) AND ('artificial intelligence'/exp OR 'artificial intelligence' OR (artificial AND ('intelligence'/exp OR intelligence)) OR 'deep learning'/exp OR 'deep learning' OR (deep AND ('learning'/exp OR learning)) OR 'transfer learning'/exp OR 'transfer learning' OR (('transfer'/exp OR transfer) AND ('learning'/exp OR learning)) OR 'machine learning'/exp OR 'machine learning' OR (('machine'/exp OR machine) AND ('learning'/exp OR learning)) OR 'deep learning system' OR (deep AND ('learning'/exp OR learning) AND system)) AND [2018-2018]/py AND 'human'/de AND 'article'/it

Method 3. Search detail of third-stage search in June 2020 for PubMed and EMBASE

3A. Search detail for PubMed

((((((((((((((((("diabetic retinopathy"[MeSH Terms] OR ("diabetic"[All Fields] AND "retinopathy"[All Fields])) OR "diabetic retinopathy"[All Fields]) OR ((((((((((("diabete"[All Fields] OR "diabetes mellitus"[MeSH Terms]) OR ("diabetes"[All Fields] AND "mellitus"[All Fields])) OR "diabetes mellitus"[All Fields]) OR "diabetes"[All Fields]) OR "diabetes insipidus"[MeSH Terms]) OR ("diabetes"[All Fields] AND "insipidus"[All Fields])) OR "diabetes insipidus"[All Fields]) OR "diabetic"[All Fields]) OR "diabetics"[All Fields]) OR "diabets"[All Fields]) AND (("macular edema"[MeSH Terms] OR ("macular"[All Fields] AND "edema"[All Fields])) OR "macular edema"[All Fields]))) OR (("macular edema"[MeSH Terms] OR ("macular"[All Fields] AND "edema"[All Fields])) OR "macular edema"[All Fields])) OR (((("retinal diseases"[MeSH Terms] OR ("retinal"[All Fields] AND "diseases"[All Fields])) OR "retinal diseases"[All Fields]) OR "retinopathies"[All Fields]) OR "retinopathy"[All Fields])) OR ((((((((((("neovascular"[All Fields] OR "neovascularisations"[All Fields]) OR "neovascularities"[All Fields]) OR "neovascularization, pathologic"[MeSH Terms]) OR ("neovascularization"[All Fields] AND "pathologic"[All Fields])) OR "pathologic neovascularization"[All Fields]) OR "neovascularisation"[All Fields]) OR "neovascularity"[All Fields]) OR "neovascularization"[All Fields]) OR "neovascularized"[All Fields]) OR "neovascularizations"[All Fields]) AND (((("retinal diseases"[MeSH Terms] OR ("retinal"[All Fields] AND "diseases"[All Fields])) OR "retinal diseases"[All Fields]) OR "retinopathies"[All Fields]) OR "retinopathy"[All Fields]))) OR ((("proliferative"[All Fields] OR "proliferatively"[All Fields]) OR "proliferatives"[All Fields]) AND (((("retinal diseases"[MeSH Terms] OR ("retinal"[All Fields] AND "diseases"[All Fields])) OR "retinal diseases"[All Fields]) OR "retinopathies"[All Fields]) OR "retinopathy"[All Fields]))) OR ("referable"[All Fields] AND (("diabetic retinopathy"[MeSH Terms] OR ("diabetic"[All Fields] AND "retinopathy"[All Fields])) OR "diabetic retinopathy"[All Fields]))) OR ((((((((((("diabete"[All Fields] OR "diabetes mellitus"[MeSH Terms]) OR ("diabetes"[All Fields] AND "mellitus"[All Fields])) OR "diabetes mellitus"[All Fields]) OR "diabetes"[All Fields]) OR "diabetes insipidus"[MeSH Terms]) OR ("diabetes"[All Fields] AND "insipidus"[All Fields])) OR "diabetes insipidus"[All Fields]) OR "diabetic"[All Fields]) OR "diabetics"[All Fields]) OR "diabets"[All Fields]) AND (((("macular edema"[MeSH Terms] OR ("macular"[All Fields] AND "edema"[All Fields])) OR "macular edema"[All Fields]) OR ("macular"[All Fields] AND "oedema"[All Fields])) OR "macular oedema"[All Fields]))) OR ((("proliferative"[All Fields] OR "proliferatively"[All Fields]) OR "proliferatives"[All Fields]) AND (("diabetic retinopathy"[MeSH Terms] OR ("diabetic"[All Fields] AND "retinopathy"[All Fields])) OR "diabetic retinopathy"[All Fields]))) OR (((("retinal diseases"[MeSH Terms] OR ("retinal"[All Fields] AND "diseases"[All Fields])) OR "retinal diseases"[All Fields]) OR ("retinal"[All Fields] AND "disorders"[All Fields])) OR "retinal disorders"[All Fields])) OR ((((((((((("diabete"[All Fields] OR "diabetes mellitus"[MeSH Terms]) OR ("diabetes"[All Fields] AND "mellitus"[All Fields])) OR "diabetes mellitus"[All Fields]) OR "diabetes"[All Fields]) OR "diabetes insipidus"[MeSH Terms]) OR ("diabetes"[All Fields] AND "insipidus"[All Fields])) OR "diabetes insipidus"[All Fields]) OR "diabetic"[All Fields]) OR "diabetics"[All Fields]) OR "diabets"[All Fields]) AND (((("eye diseases"[MeSH Terms] OR ("eye"[All Fields] AND "diseases"[All Fields])) OR "eye diseases"[All Fields]) OR ("eye"[All Fields] AND "disease"[All Fields])) OR "eye disease"[All Fields]))) OR ((("blindness"[MeSH Terms] OR "blindness"[All Fields]) OR ("vision"[All Fields] AND "loss"[All Fields])) OR "vision loss"[All Fields])) OR (("retinal diseases"[MeSH Terms] OR ("retinal"[All Fields] AND "diseases"[All Fields])) OR "retinal diseases"[All Fields])) OR ("macular"[All Fields] AND (((("disease"[MeSH Terms] OR "disease"[All Fields]) OR "diseases"[All Fields]) OR "disease s"[All Fields]) OR "diseased"[All Fields]))) OR (("macular degeneration"[MeSH Terms] OR ("macular"[All Fields] AND "degeneration"[All Fields])) OR "macular degeneration"[All Fields])) OR ("macular"[All Fields] AND ((((("disease"[MeSH Terms] OR "disease"[All Fields]) OR "disorder"[All Fields]) OR "disorders"[All Fields]) OR "disorder s"[All Fields]) OR "disordes"[All Fields]))) AND (((((("artificial intelligence"[MeSH Terms] OR ("artificial"[All Fields] AND "intelligence"[All Fields])) OR "artificial intelligence"[All Fields]) OR (("deep learning"[MeSH Terms] OR ("deep"[All Fields] AND "learning"[All Fields])) OR "deep learning"[All Fields])) OR (((("machine learning"[MeSH Terms] OR ("machine"[All Fields] AND "learning"[All Fields])) OR "machine learning"[All Fields]) OR ("transfer"[All Fields] AND "learning"[All Fields])) OR "transfer learning"[All Fields])) OR (("machine learning"[MeSH Terms] OR ("machine"[All Fields] AND "learning"[All Fields])) OR "machine learning"[All Fields])) OR ((("deep learning"[MeSH Terms] OR ("deep"[All Fields] AND "learning"[All Fields])) OR "deep learning"[All Fields]) AND ((((("drug delivery systems"[MeSH Terms] OR (("drug"[All Fields] AND "delivery"[All Fields]) AND "systems"[All Fields])) OR "drug delivery systems"[All Fields]) OR "system"[All Fields]) OR "system s"[All Fields]) OR "systems"[All Fields])))

3B. Search detail for EMBASE

('diabetic retinopathy'/exp OR 'diabetic retinopathy' OR (('diabetic'/exp OR diabetic) AND ('retinopathy'/exp OR retinopathy)) OR 'diabetic macular edema'/exp OR 'diabetic macular edema' OR (('diabetic'/exp OR diabetic) AND macular AND ('edema'/exp OR edema)) OR 'macular edema'/exp OR 'macular edema' OR (macular AND ('edema'/exp OR edema)) OR 'retinopathy'/exp OR retinopathy OR 'neovascularized retinopathy' OR (neovascularized AND ('retinopathy'/exp OR retinopathy)) OR 'proliferative retinopathy'/exp OR 'proliferative retinopathy' OR (proliferative AND ('retinopathy'/exp OR retinopathy)) OR 'referable diabetic retinopathy' OR (referable AND ('diabetic'/exp OR diabetic) AND ('retinopathy'/exp OR retinopathy)) OR 'diabetic macular oedema'/exp OR 'diabetic macular oedema' OR (('diabetic'/exp OR diabetic) AND macular AND ('oedema'/exp OR oedema)) OR 'proliferative diabetic retinopathy'/exp OR 'proliferative diabetic retinopathy' OR (proliferative AND ('diabetic'/exp OR diabetic) AND ('retinopathy'/exp OR retinopathy)) OR 'retinal disorders' OR (('retinal'/exp OR retinal) AND ('disorders'/exp OR disorders)) OR 'diabetic eye disease'/exp OR 'diabetic eye disease' OR (('diabetic'/exp OR diabetic) AND ('eye'/exp OR eye) AND ('disease'/exp OR disease)) OR 'vision loss'/exp OR 'vision loss' OR (('vision'/exp OR vision) AND loss) OR 'retinal diseases'/exp OR 'retinal diseases' OR (('retinal'/exp OR retinal) AND ('diseases'/exp OR diseases)) OR 'macular disease'/exp OR 'macular disease' OR (macular AND ('disease'/exp OR disease)) OR 'macular degeneration'/exp OR 'macular degeneration' OR (macular AND ('degeneration'/exp OR degeneration)) OR 'macular disorders'/exp OR 'macular disorders' OR (macular AND ('disorders'/exp OR disorders))) AND ('artificial intelligence'/exp OR 'artificial intelligence' OR (artificial AND ('intelligence'/exp OR intelligence)) OR 'deep learning'/exp OR 'deep learning' OR (deep AND ('learning'/exp OR learning)) OR 'transfer learning'/exp OR 'transfer learning' OR (('transfer'/exp OR transfer) AND ('learning'/exp OR learning)) OR 'machine learning'/exp OR 'machine learning' OR (('machine'/exp OR machine) AND ('learning'/exp OR learning)) OR 'deep learning system' OR (deep AND ('learning'/exp OR learning) AND system)) AND [2018-2020]/py AND 'human'/de AND 'article'/it AND [16-12-2018]/sd NOT [8-6-2020]/sd AND [article]/lim AND [humans]/lim AND [english]/lim
