# Supplementary material for: Performance and Limitation of Machine Learning Algorithms for Diabetic Retinopathy Screening: Meta-analysis
Source: J Med Internet Res. 2021 Jul 5;23(7):e23863. doi: 10.2196/23863 (PMC8406115; doi:10.2196/23863)
Supplement: Multimedia Appendix 2 [file jmir_v23i7e23863_app2.docx]

| **Study** | **Year** | **Country** | **Algorithms** | **ML category** | **Source of testing patients or database** | **Goal of detection** | **Reference standard** |
| --- | --- | --- | --- | --- | --- | --- | --- |
| Chaum E et al | 2008 | USA | Supervised learning | Others | University of Tennessee Hamilton Eye Institute | PDR | 1 graders |
| Agurto C et al | 2011 | New Mexico | K-means clustering and 2-step partial least squares regression | Others | Retina Institute of South Texas | DR, VTDR | 2 graders |
|  |  |  |  |  | University of Texas Health Science Center in San Antonio |  |  |
| Ting DSW et al | 2017 | Singapore | Deep neural network | NN | Singapore National Diabetic Retinopathy Screening Program, 2014-2015 | VTDR, mtmDR | 3 graders |
|  |  |  |  |  | Guangdong community based dataset | mtmDR | 2 graders |
|  |  |  |  |  | Singapore Malay Eye Study |  |  |
|  |  |  |  |  | Singapore Indian Eye Study |  |  |
|  |  |  |  |  | Singapore Chinese Eye Study |  |  |
|  |  |  |  |  | Beijing Eye Study |  |  |
|  |  |  |  |  | African American Eye Disease Study |  |  |
|  |  |  |  |  | Royal Victoria Eye and Ear Hospital |  |  |
|  |  |  |  |  | Mexican dataset |  |  |
|  |  |  |  |  | Chinese University of Hong Kong |  |  |
|  |  |  |  |  | University of Hong Kong |  |  |
| Quellec G et al | 2012 | France | Instance Learning | Others | OPHDIAT study of EyePACs | mtmDR | Medical records |
| Rajalakshmi R  et al | 2018 | India | EyeArtTM | NN | Tertiary care diabetes hospital in Chennai, Southern India | DR, VTDR | More than 2 graders |
| Gargeya R et al | 2017 | USA | Deep feature learning and Decision Tree Classification Model | NN | MESSIDOR-2 database | DR | Benchmark database |
| Pires R et al | 2015 | Australia | Bag of Visual Words | Others | Inala Aboriginal and Torres Strait Islander health care centre | DR | 3 graders |
| Jelinek HF et al | 2007 | Australia | Linear discriminant analysis classifier | Others | N.A. | PDR | N.A. |
| Gardner GG  et al | 1996 | Scotland | Back-propagation neural network | NN | N.A. | DR | 1 grader |
| Abbas Q et al | 2017 | Saudi Arabia | Semi-supervised multilayer deep-learning algorithm | NN | DIARETDB1. FAZ, MESSIDOR, Private Hospital Universitario Puerta del Mar (Prv-DR) datasets | PDR | 2 graders |
| Quellec G et al | 2018 | France | ConvNets | NN | DIARETDB1 dataset | DR | Benchmark database |
|  |  |  |  |  | Kaggle-test dataset | mtmDR |  |
| Usman Akram M et al | 2013 | Pakistan | Multivariate m-Mediods based classifier | Others | DRIVE dataset | PDR | Benchmark database |
|  |  |  |  |  | STARE dataset |  |  |
|  |  |  |  |  | DIARETDB dataset |  |  |
|  |  |  |  |  | MESSIDOR dataset |  |  |
| Gulshan V et al | 2016 | USA | Deep learning algorithm | NN | EyePACS screening site | VTDR, mtmDR | More than 2 graders |
|  |  |  |  |  | MESSIDOR-2 dataset | mtmDR |  |
| Raju M et al | 2017 | India | Convolution neural network | NN | EyePACS dataset | DR, PDR | Benchmark database |
| Annie Grace Vimala GS et al | 2017 | India | BF-kernel based Support Vector Machine | SVM | One eye care center in Chennai (India) | DR | 1 grader |
|  |  |  |  |  |  |  | Benchmark database |
|  |  |  |  |  | DIARETDB1 dataset |  |  |
| Welikala RA et al | 2015 | UK | Linear-support vector machine classifier | SVM | MESSIDOR, St Thomas’ Hospital ophthalmology department | PDR | Benchmark database and  1 grader |
| Gupta G et al | 2017 | India | Random Forest classifier | RF | Local database from  4 different clinical centres, MESSIDOR, STARE, High resolution fundus (HRF) datasets | PDR | Medical records and  2 graders |
|  |  |  |  |  | MESSIDOR, STARE, HRF datasets |  |  |
|  |  |  |  |  | HRF dataset |  |  |
|  |  |  |  |  | Local database |  |  |
|  |  |  |  |  | STARE dataset |  |  |
| Orlando JI et al | 2017 | Argentina | Regularized logistic regulation of binary classification | Others | MESSIDOR dataset | PDR | 2 graders |
| Zhang Y et al | 2016 | China | Active Learning with Query by Committee | Others | MESSIDOR dataset | DR | Benchmark database |
| Ganesan K et al | 2014 | Singapore | Probabilistic neural network and Genetic algorithm | NN | MESSIDOR dataset | DR | Benchmark database |
|  |  |  |  |  | Department of ophthalmology, Kasturba Medical College, Manipal, India |  | N.A. |
| Bala MP et al | 2015 | India | Extreme Learning Machine | Others | DIARETDB0 dataset | PDR | Benchmark databases |
|  |  |  |  |  | DRIVE dataset |  |  |
| Abramoff MD et al | 2016 | USA | IDx-DR X2.1 | NN | MESSIDOR-2 dataset | VTDR, mtmDR | Benchmark database |
| Orlando JI et al | 2017 | Argentina | Convolutional neural network and Random forest classifier | Others | MESSIDOR dataset | DR | Benchmark databases |
|  |  |  |  |  | e-Ophtha dataset |  |  |
| Adal KM et al | 2014 | France | Semi-supervised learning model: Support Vector Machines-co trained | SVM | University of Texas Health Science Center in San Antonio | DR | Multiple graders |
|  |  |  | Semi-supervised learning model: k-nearest neighbor-co trained | Others | DIARETDB1 dataset |  | Benchmark database |
| Sangeethaa SN et al | 2018 | India | Convolutional neural network | NN | DRIVE, DIARETDB0,DIARETDB1_v1, Aravind Eye Hospital (India) datasets | DR | Benchmark  databases and graders |
| Li Z, 2018 et al | 2018 | China | Convolutional neural network | NN | National Indigenous Eye Health Survey, Singapore Malay Eye Study, Australian Diabetes Obesity and Lifestyle Study | VTDR | 3 graders |
| Sumathy B et al | 2018 | India | Back propagation neural network | NN | Rajiv Gandhi Eye hospital from Trichy | DR | N.A. |
| Fadafen MK et al | 2018 | Iran | Computational model of Human Visual System | Others | DIARETDB1 dataset | DR | Benchmark  database |
| Yu S, 2018 et al | 2018 | Australia | Support vector machine | SVM | MESSIDOR, HRF, DIARETDB0, Kaggle diabetic retinopathy datasets | PDR | Benchmark  databases |
| Ramachandran N et al | 2018 | New Zealand | Visiona | NN | ODEMS DR screening photos | mtmDR | 2 graders |
|  |  |  |  |  | MESSIDOR dataset |  | Benchmark  database |
| Malathi K et al | 2018 | India | Recursive Support Vector Machine: Shrinking Edge-Mark model | SVM | Local hospitals | DR | N.A. |
| Abràmoff MD et al | 2018 | USA | Multilayer convolutional neural networks | NN | Subjects enrolled at 10 sites for pivotal study | mtmDR | Wisconsin Fundus Photograph Reading Center |
| Stevenson CH et al | 2019 | New Zealand | Convolutional neural networks | NN | Multiple publicly available fundus datasets | DR | Benchmark  databases |
| Son J et al | 2020 | Korea | Convolutional neural networks | NN | Seoul National University Bundang Hospital Retina Image Archive | DR | 3 graders |
|  |  |  |  |  | e-ophtha EX dataset |  | Benchmark  databases |
|  |  |  |  |  | Indian Diabetic Retinopathy Image Dataset (IDRiD) |  |  |
|  |  |  |  |  | MESSIDOR dataset |  |  |
| Verbraak FD et al | 2019 | Netherlands | IDx-DR-EU-2.1 | NN | Star-SHL, Rotterdam (Netherlands) | mtmDR  VTDR | 2 graders (Rotterdam Study reading center) |
| Pires R et al | 2019 | Brazil | Deep convolutional neural network | NN | EyePACS (Kaggle) training set | mtmDR | Benchmark  databases |
|  |  |  |  |  | MESSIDOR-2 dataset. |  |  |
|  |  |  | RF+ transfer learning | RF | DR2 dataset (Department of Ophthalmology, Federal University of São Paulo) |  |  |
| Voets M et al | 2019 | Norway | InceptionV3 model | NN | MESSIDOR-2 dataset. | mtmDR | Benchmark  database |
| Chowdhury AR et al | 2019 | India | Naïve Bayes | Others | DIARETDB0, DIARETDB1, Tele-ophtha, MESSIDOR, HRF, Retinal image computing and understanding (University of Lincoln), other datasets | DR | Benchmark  databases |
| Long S et al | 2019 | China | fuzzy C-means clustering + Support vector machine | SVM | DIARETDB1 dataset | DR | Benchmark  database |
| Khojasteh P et al | 2019 | Australia | ResNet-50+Support vector machine | Others | DIARETDB1 dataset | DR | Benchmark  databases |
|  |  |  |  |  | e-Ophtha EX dataset |  |  |
| Ullah H et al | 2019 | Pakistan | Multi-Layer Feed Forward Perceptron + Chain-like agent genetic algorithm | NN | DIARETDB0, DIARETDB1, DRIVE,  Al-Shifa Trust Eye Hospital datasets | DR | Benchmark and hospital  Databases |
| Wang H et al | 2020 | China | Deep convolutional neural network +Random forest | Others | e-Ophtha EX dataset | DR | Benchmark databases |
|  |  |  |  |  | HEI-MED datasets |  |  |
| Xie L et al | 2020 | New Zealand | Diabetic Retinopathy classifier convolutional neural network | NN | EyePACS (Kaggle) dataset | DR  mtmDR | Benchmark database |
| He J et al | 2020 | China | Inception V4 model | NN | PengPu Town Community Hospital | DR  mtmDR | 2 graders |
| Colomer A et al | 2020 | Spain | Gaussian processes for classification | Others | e-Ophtha EX dataset | DR | Benchmark databases |
|  |  |  |  |  | DIARETDB1 dataset |  |  |
| Sosale B et al | 2020 | India | Medios AI algorithm | NN | Outpatient department of Diacon Hospital, Bangalore (India) | DR  mtmDR | 4 graders |
| Shah P et al | 2020 | Singapore | Deep convolutional neural network | NN | Sankara Eye Hospital | DR  mtmDR  VTDR  PDR | 2 graders |
|  |  |  |  |  | MESSIDOR dataset |  | Benchmark database |
| Zago GT et al | 2020 | Brazil | VCG 16 model + Transfer learning | NN | MESSIDOR dataset | DR | Benchmark databases |
|  |  |  |  |  | EyePACS (Kaggle) training set |  |  |
|  |  |  |  |  | IDRiD dataset |  |  |
|  |  |  |  |  | DDR dataset |  |  |
|  |  |  |  |  | DIARETDB0 dataset |  |  |
| Riaz H et al | 2020 | Korea | DenseNets | NN | MESSIDOR-2 dataset. | DR  mtmDR  VTDR  PDR | Benchmark databases |
|  |  |  |  |  | EyePACS (Kaggle) training set | DR  mtmDR  VTDR  PDR |  |
| Raumviboonsuk P et al | 2019 | India | Deep learning model | NN | National screening program for DR, Ministry of Public Health of Thailand | mtmDR  VTDR | Medical  records |
| Li F et al | 2019 | China | Inception-v3 network + Transfer learning | NN | MESSIDOR-2 dataset. | mtmDR | Benchmark database |
| Bhaskaranand M et al | 2019 | USA | EyeArt system v2.0 | NN | EyePACS DR telescreening program (Jan 2014- Sep 2015) | mtmDR | Medical  records |
| Yang WH et al | 2019 | China | VGGNet + transfer learning | NN | Intelligent Ophthalmology Database of Zhejiang Society for Mathematical Medicine, China. | DR  mtmDR  VTDR | Medical records |
| Natarajan S et al | 2019 | India | Medios AI algorithm | NN | Community patients visits (Municipal Corporation of Greater Mumbai, India) | DR  mtmDR | 2 graders |
| Gulshan V et al | 2019 | India | Deep neural networks | NN | Aravind EyeHospital | mtmDR | Multiple graders |
|  |  |  |  |  | Sankara Nethralaya |  |  |
| Nazir T et al | 2019 | Pakistan | Extreme learning machine | NN | DRIVE dataset | DR | Benchmark databases |
|  |  |  |  |  | STARE dataset |  |  |
| Bellemo V et al | 2019 | Singapore | Adapted VGGNet architecture + Residual neural network architecture | NN | 5 urban centres in the Copperbelt province of Zambia (Feb - Jun 2012) | mtmDR  VTDR | 2 graders |
| Badgujar RD | 2019 | India | Hybrid SMO-GBM classifier. | Others | STARE dataset | DR | Benchmark database |
| Hemanth DJ et al | 2018 | India | Modified Hopfield Neural Network | NN | Lotus Eye Care Hospital, India | DR | Medical records |
| Kanagasingam Y | 2018 | Australia | Neural network + Transfer-learning | NN | Primary care practice in Midland, Western Australia (Dec 2016- May 2017) | mtmDR | 4 graders |

^a^Abbreviation: Diabetic retinopathy=DR, Proliferative diabetic retinopathy=PDR, Vision-threatening diabetic retinopathy=VTDR, More-than-mild diabetic retinopathy=mtmDR, N.A.=Not applicable (not reported), NN=Neural network, SVM =Support vector machine, RF=Random forest
