# Supplementary material for: Performance and Limitation of Machine Learning Algorithms for Diabetic Retinopathy Screening: Meta-analysis
Source: J Med Internet Res. 2021 Jul 5;23(7):e23863. doi: 10.2196/23863 (PMC8406115; doi:10.2196/23863)
Supplement: Multimedia Appendix 3 [file jmir_v23i7e23863_app3.docx]

| **Database** | **No. of images** | **Patient population** | **Source of images** | **FOV** | **Pixels of image** | **Image Composition** | **Groundtruth information** | **Studies applying the database for validation** |
| --- | --- | --- | --- | --- | --- | --- | --- | --- |
| MESSIDOR | 1200 | DM patients | Three ophthalmologic departments in France | 45^o^ | 1440*960, 2240*1488, 2304*1536 | 546 normal images and 654 images with DR | Retinopathy grade, risk of macular edema | Abbas Q, et al. 2017  Usman Akram M, et al. 2013  Gulshan V, et al. 2016  Welikala RA, et al. 2015  Gupta G, et al. 2017  Orlando JI, et al. 2017  Zhang Y, et al. 2016  Ganesan K, et al. 2014  Orlando JI, et al. 2017  Yu S, et al. 2018  Ramachandran N, et al. 2018  Stevenson CH, et al. 2019  Son J, et al. 2020  Shah P, et al. 2020  Chowdhury AR, et al. 2019 |
| MESSIDOR-2 | 1784 | DM patients | Brest University Hospital, France | 45^o^ | 1440*960, 2240*1488, 2304*1536 | 1017 normal images and 731 images with DR | Retinopathy grade, risk of macular edema | Gargeya R, et al. 2017  Abramoff MD, et al. 2016  Pires R, et al. 2020  Voets M, et al. 2019  Riaz H, et al. 2020  Li F, et al. 2019 |
| STARE | 400 | patients with variable ophthalmic diseases | Shiley Eye Center, University of California, San Diego, USA | 35^o^ | 700*605 | 41 normal images and 91 images with DR | Diagnosis, annotations of visible features,  hand labels of vessels and optic nerve | Usman Akram M, et al. 2013  Gupta G, et al. 2017  Stevenson CH, et al. 2019  Nazir T, et al. 2019  Badgujar RD, et al. 2019 |
| DRIVE | 40 | DM patients | Retinopathy screening program in the Netherlands | 45^o^ | 768*584 | 33 normal images and 7 images with signs of mild early DR | Manual segmentation of vasculatures | Usman Akram M, et al. 2013  Bala MP, et al. 2015  Sangeethaa SN, et al. 2018  Stevenson CH, et al. 2019  Ullah H, et al. 2019 |
| e-Ophtha | 463 | DM patients | The OPHDIAT network, France | 40^o^ | 2544*1696  1440*960 | e-Ophtha_EX: 82 images in total; 35 normal images and 47 images with exudates.  e-Ophtha_MA: 381 images in total; 233 normal images and 148 images with microaneurysms or small hemorrhage. | Manual markings of DR lesions | Orlando JI, et al. 2017  Stevenson CH, et al. 2019  Son J, et al. 2020  Khojasteh P, et al. 2020  Wang H, et al. 2020  Colomer A, et al. 2020 |
| DIARETDB0 | 130 | DM patients | Kuopio university hospital, Finland | 50^o^ | 1500*1152 | 20 normal images and 110 images with signs of DR | NA | Usman Akram M, et al. 2013  Bala MP, et al. 2015  Sangeethaa SN, et al. 2018  Yu S, et al. 2018  Stevenson CH, et al. 2019  Chowdhury AR, et al. 2019  Ullah H, et al. 2019  Zago GT, et al. 2020 |
| DIARETDB1 | 89 | DM patients | Kuopio university hospital, Finland | 50^o^ | 1500*1152 | 5 normal images and 84 images with signs of at least mild non-proliferative DR | Manual markings of signs of DR | Abbas Q, et al. 2017  Quellec G, et al. 2018  Usman Akram M, et al. 2013  Annie Grace Vimala GS, et al. 2017  Adal KM, et al. 2014  Sangeethaa SN, et al. 2018  Ullah H, et al. 2019  Fadafen MK, et al. 2018  Stevenson CH, et al. 2019  Chowdhury AR, et al. 2019  Long S, et al. 2019  Khojasteh P, et al. 2019  Colomer A, et al. 2020 |
| EyePACS (Kaggle-DR) | 88704 | NA | EyePACS DR screening platform, USA | 45o | 5184*3456  3888*2592 | 65300 normal images and 23400 images with sings of DR | Retinopathy grade | Raju M, et at. 2017  Gulshan V, et al. 2016  Pires R, et al. 2019  Xie L, et al. 2020  Zago GT, et al. 2020  Riaz H, et al. 2020  Bhaskaranand M, et al. 2019 |

^a^Abbreviation: No.=Number, DM=Diabetes mellitus, DR=Diabetic retinopathy, FOV=Field of view
