# Supplementary material for: Performance and Limitation of Machine Learning Algorithms for Diabetic Retinopathy Screening: Meta-analysis
Source: J Med Internet Res. 2021 Jul 5;23(7):e23863. doi: 10.2196/23863 (PMC8406115; doi:10.2196/23863)
Supplement: Multimedia Appendix 4 [file jmir_v23i7e23863_app4.docx]

| **Methods/algorithms** | **Description** |
| --- | --- |
| Machine learning algorithm | Machine learning is a field that utilizes computers to build a model to learn patterns from training data and make predictions, and the models that computers use to progressively improve performance on specific tasks are called “algorithms”. |
| Random forest | An ensemble method that constructs the results of multiple decision trees to avoid bias and overfitting. |
| Supportive vector machine | A type of learning method that utilizes labeled training data to build an optimal hyperplane in a high-dimensional space with dividing structure that helps classification of new data points. The form of the dividing structure is dependent on the dimension of the space, for example, it is usually a line in a 2 dimensional space. |
| Neural network | A type of machine learning algorithms that is structurally similar to biological brain with the basic functional unit called “neuron”. The neurons are typically aggregated into layers, and the most common network structure is composed of one input layer, one output layer, and one or multiple hidden layers. Each edge in the network has a weight, and each neuron of the hidden and output layers has a function that calculates values fed by previous layers. |
| Deep neural network | A type of neural network with multiple hidden layers besides the input and output layers. This type of network is usually a feedforward network, and is commonly applied on image recognition. |
| Convolutional neural network | A class of deep neural network with a special mathematical operation called “convolution” that is useful for image recognition. It usually contains three types of layers: convolution layers, which calculate values from the original image with a specific feature detector; pooling layers, which compress the calculated values and keep the critical information; fully connected layers, which flattens the value from previous layers and connect itself to another layer. |
| Transfer learning | A new type of algorithm that applies a trained neural network model to another domain. By replacing the last output layer of the previous model with a new one and training the hidden layers retrogradely, this algorithm “transfers” the previously trained structure to a new model that conducts a new task. The advantages of transfer learning is reduced cost of training and applicability on target domain with insufficient data. |
