# Supplementary material for: Performance and Limitation of Machine Learning Algorithms for Diabetic Retinopathy Screening: Meta-analysis
Source: J Med Internet Res. 2021 Jul 5;23(7):e23863. doi: 10.2196/23863 (PMC8406115; doi:10.2196/23863)

Figure S1. Summary of QUADAS-2 assessment for included studies

Figure S1a. Risk of bias

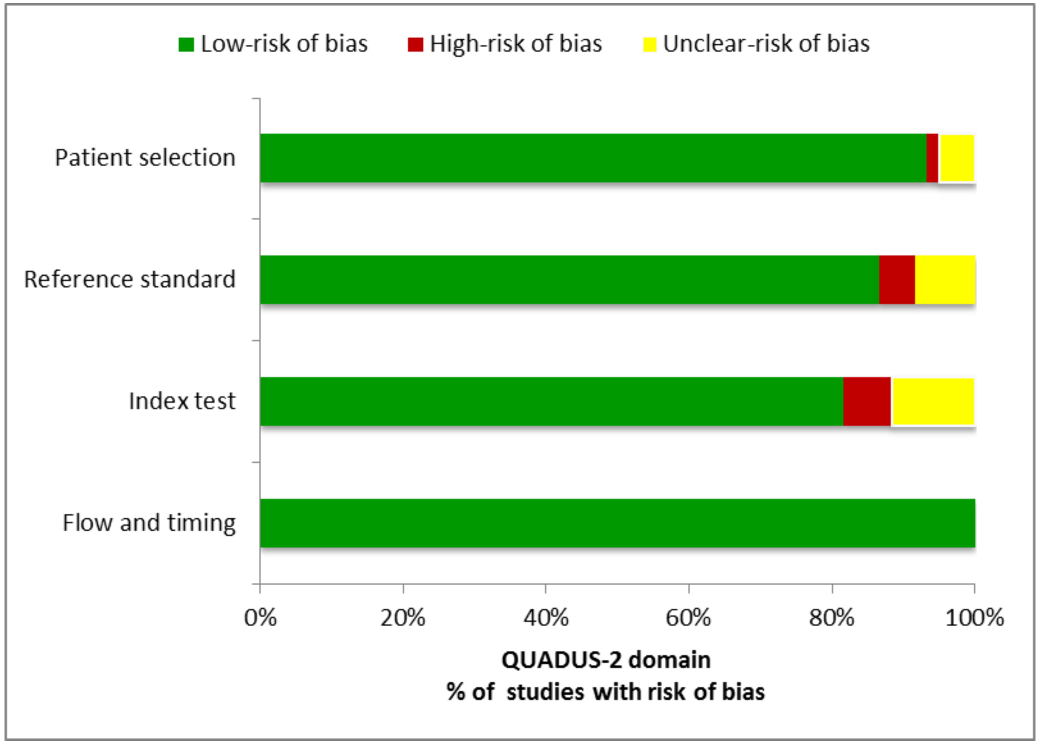

Figure S1b. Concern of applicability

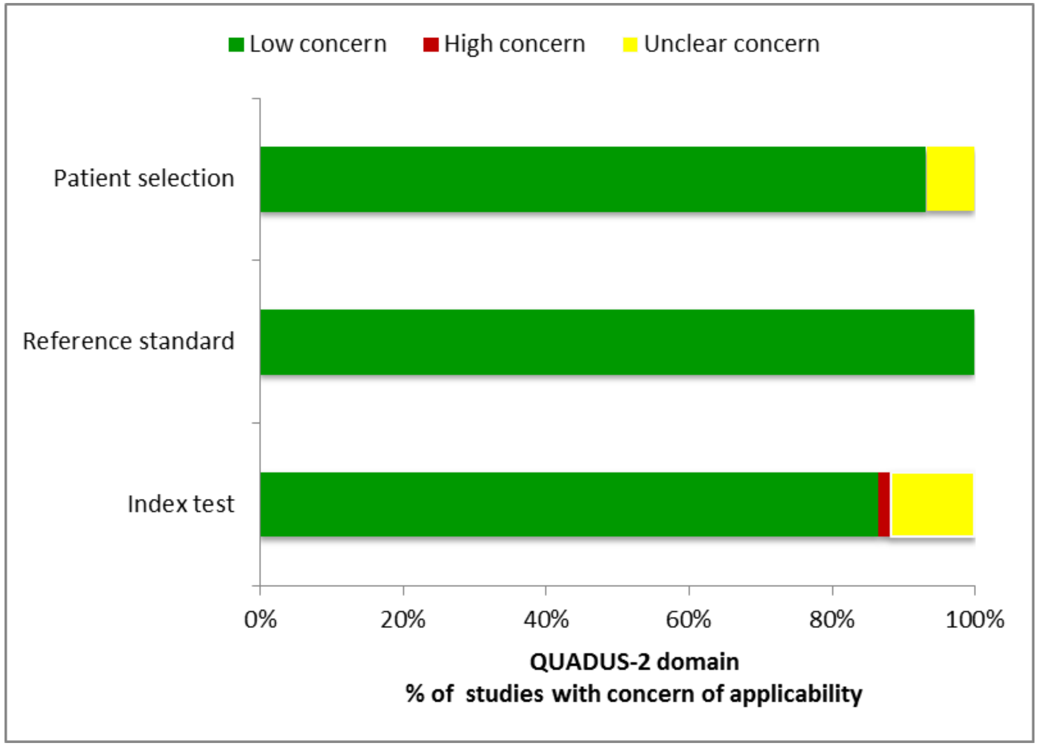

Supplement: Multimedia Appendix 5 [file jmir_v23i7e23863_app5.pdf]
