# Supplementary material for: Performance and Limitation of Machine Learning Algorithms for Diabetic Retinopathy Screening: Meta-analysis
Source: J Med Internet Res. 2021 Jul 5;23(7):e23863. doi: 10.2196/23863 (PMC8406115; doi:10.2196/23863)
Supplement: Multimedia Appendix 6 [file jmir_v23i7e23863_app6.docx]

| **Study** | **Year** | **Country** | **Algorithms** | **ML category** | **Goal of detection** | **Source of database** | **Sample size (n)** | **No. with findings (%)** | **Sen** | **Spe** |
| --- | --- | --- | --- | --- | --- | --- | --- | --- | --- | --- |
| Ting DSW et al | 2017 | Singapore | Deep neural network | NN | VTDR | Singapore National Diabetic Retinopathy Screening Program | 35958 | 514 (1.4) | 1.00 | 0.92 |
|  |  |  |  |  | mtmDR | Singapore National Diabetic Retinopathy Screening Program | 35958 | 969 (2.7) | 0.91 | 0.92 |
|  |  |  |  |  |  | Guangdong community | 7988 | 891 (11.2) | 0.99 | 0.82 |
|  |  |  |  |  |  | Singapore Malay Eye Study | 1256 | 193 (15.4) | 0.97 | 0.82 |
|  |  |  |  |  |  | Singapore Indian Eye Study | 2256 | 218 (96.6) | 0.99 | 0.73 |
|  |  |  |  |  |  | Singapore Chinese Eye Study | 968 | 85 (8.8) | 1.00 | 0.76 |
|  |  |  |  |  |  | Beijing Eye Study | 526 | 27 (5.1) | 0.94 | 0.89 |
|  |  |  |  |  |  | African American Eye Disease Study | 968 | 86 (8.9) | 0.99 | 0.87 |
|  |  |  |  |  |  | Royal Victoria Eye and Ear Hospital | 1151 | 722 (62.7) | 0.99 | 0.92 |
|  |  |  |  |  |  | Mexican population | 586 | 484 (82.6) | 0.92 | 0.85 |
|  |  |  |  |  |  | Chinese University of Hong Kong | 627 | 385 (61.4) | 0.99 | 0.83 |
|  |  |  |  |  |  | University of Hong Kong | 3853 | 383 (9.9) | 1.00 | 0.81 |
| Chaum E et al | 2008 | USA | Supervised learning | Others | PDR | Tennessee Hamilton Eye Institute | 395 | 14 (3.5) | 1.00 | 0.98 |
| Agurto C et al | 2011 | New Mexico | K-means clustering and 2-step partial least squares regression | Others | DR | Retina Institute of South Texas | 563 | 419 (74.4) | 0.92 | 0.60 |
|  |  |  |  |  |  | University of Texas Health Science Center | 573 | 437 (76.3) | 0.94 | 0.60 |
|  |  |  |  |  | VTDR | Retina Institute of South Texas | 337 | 193 (57.3) | 0.95 | 0.60 |
|  |  |  |  |  |  | University of Texas Health Science Center | 449 | 313 (69.7) | 0.96 | 0.60 |
| Pires R et al | 2015 | Australia | Bag of Visual Words | Others | DR | Inala Aboriginal and Torres Strait Islander health care centre | 30 | 12 (20.0) | 1.00 | 0.89 |
| Jelinek HF et al | 2007 | Australia | Linear discriminant analysis classifier | Others | DR | Unknown dataset | 27 | 16 (59.3) | 0.94 | 0.82 |
| Gardner GG et al | 1996 | Scotland | Back-propagation neural network | NN | DR | Unknown dataset | 301 | 200 (66.4) | 0.88 | 0.84 |
| Abbas Q et al | 2017 | Saudi Arabia | Semi-supervised multilayer deep-learning algorithm | NN | PDR | DIARETDB1. FAZ, MESSIDOR, Private Hospital Universitario Puerta del Mar datasets | 750 | 150 (20.0) | 0.88 | 0.92 |
| Quellec G et al | 2018 | France | ConvNets | NN | DR | DIARETDB1 dataset | 89 | 84 (94.4) | 0.90 | 0.99 |
|  |  |  |  |  | mtmDR | EyePACS (Kaggle) test dataset | 53576 | 10501 (19.6) | 0.88 | 0.93 |
| Usman Akram M et al | 2013 | Pakistan | Multivariate m-Mediods based classifier | Others | PDR | DRIVE dataset | 40 | 3 (7.5) | 1.00 | 1.00 |
|  |  |  |  |  |  | STARE dataset | 81 | 5 (6.2) | 0.93 | 0.98 |
|  |  |  |  |  |  | DIARETDB dataset | 89 | 7 (7.9) | 0.90 | 0.98 |
|  |  |  |  |  |  | MESSIDOR dataset | 1200 | 37 (3.1) | 0.98 | 0.97 |
| Gulshan V et al | 2016 | USA | Deep learning algorithm | NN | VTDR | EyePACS-1 dataset | 8788 | 149 (1.7) | 0.84 | 0.99 |
|  |  |  |  |  | mtmDR | EyePACS-1 dataset | 8788 | 683 (7.8) | 0.98 | 0.93 |
|  |  |  |  |  |  | MESSIDOR-2 dataset | 1745 | 254 (14.6) | 0.96 | 0.94 |
| Raju M et al | 2017 | India | Convolution neural network | NN | DR | EyePACS (Kaggle) test dataset | 53576 | 13593 (25.3) | 0.89 | 0.92 |
|  |  |  |  |  | PDR |  | 53576 | 1206 (2.3) | 0.53 | 1.00 |
| Annie Grace Vimala GS et al | 2017 | India | BF-kernel based Support Vector Machine | SVM | DR | One eye care center in Chennai, India | 24 | 14 (58.3) | 0.92 | 0.91 |
|  |  |  |  |  |  | DIARETDB1 dataset | 36 | 34 (94.4) | 0.92 | 0.90 |
| Welikala RA et al | 2015 | UK | Linear-support vector machine classifier | SVM | PDR | MESSIDOR, St Thomas’ Hospital ophthalmology department | 60 | 20 (33.3) | 1.00 | 0.93 |
| Gupta G et al | 2017 | India | Random Forest classifier | RF | PDR | Local database from 4 different clinical centres, MESSIDOR, STARE, HRF datasets | 779 | 100 (12.8) | 0.92 | 0.93 |
|  |  |  |  |  |  | MESSIDOR, STARE, HRF datasets | 139 | 11 (7.9) | 1.00 | 0.92 |
|  |  |  |  |  |  | HRF dataset | 20 | 5 (25.0) | 0.96 | 0.92 |
|  |  |  |  |  |  | Local database | 518 | 74 (14.3) | 0.97 | 0.92 |
|  |  |  |  |  |  | STARE dataset | 73 | 10 (13.7) | 0.93 | 0.93 |

| **Study** | **Year** | **Country** | **Algorithms** | **ML category** | **Goal of detection** | **Source of database** | **Sample size (n)** | **No. with findings (%)** | **Sen** | **Spe** |
| --- | --- | --- | --- | --- | --- | --- | --- | --- | --- | --- |
| Orlando JI et al | 2017 | Argentina | Regularized logistic regulation of binary classification | Others | PDR | MESSIDOR dataset | 59 | 15 (25.4) | 0.85 | 0.89 |
| Zhang Y et al | 2016 | China | Active Learning with Query by Committee | Others | DR | MESSIDOR dataset | 173 | 81 (46.8) | 0.83 | 0.92 |
| Ganesan K et al | 2014 | Singapore | Probabilistic neural network and Genetic algorithm | NN | DR | MESSIDOR dataset | 340 | 170 (50.0) | 1.00 | 1.00 |
|  |  |  |  |  |  | Department of ophthalmology, Kasturba Medical College | 340 | 170 (50.0) | 0.99 | 0.99 |
| Bala MP et al | 2015 | India | Extreme Learning Machine | Others | PDR | DIARETDB0 dataset | 130 | 30 (23.1) | 0.97 | 1.00 |
|  |  |  |  |  |  | DRIVE dataset | 40 | 7 (17.5) | 1.00 | 0.94 |
| Abramoff MD et al | 2016 | USA | IDx-DR X2.1 | NN | VTDR | MESSIDOR-2 dataset | 874 | 93 (10.6) | 1.00 | 0.91 |
|  |  |  |  |  | mtmDR |  | 874 | 200 (22.9) | 0.97 | 0.87 |
| Orlando JI et al | 2017 | Argentina | Convolutional neural network and Random forest classifier | Others | DR | MESSIDOR dataset | 1200 | 654 (54.5) | 0.91 | 0.50 |
|  |  |  |  |  |  | e-Ophtha dataset | 381 | 148 (38.8) | 0.78 | 0.90 |
| **Study** | **Year** | **Country** | **Algorithms** | **ML category** | **Goal of detection** | **Source of database** | **Sample size (n)** | **No. with findings (%)** | **Sen** | **Spe** |
| Adal KM et al | 2014 | France | Semi-supervised learning model: Support Vector Machines-co trained | SVM | DR | UTHSC dataset | 50 | 37 (74.0) | 0.81 | 0.92 |
|  |  |  | Semi-supervised learning model: k-nearest neighbor-co trained | Others |  | DIARETDB1 dataset | 89 | 84 (94.4) | 0.64 | 0.79 |
| Sangeethaa SN et al | 2018 | India | Convolutional neural network | NN | DR | DRIVE, DIARETDB0,DIARETDB1_v1, Aravind Eye Hospital datasets | 292 | 217 (74.3) | 0.98 | 0.93 |
| Li Z, 2018 et al | 2018 | China | Convolutional neural network | NN | VTDR | National Indigenous Eye Health Survey, Singapore Malay Eye Study, Australian Diabetes Obesity and Lifestyle Study | 13657 | 401 (2.9) | 0.93 | 0.99 |
| **Study** | **Year** | **Country** | **Algorithms** | **ML category** | **Goal of detection** | **Source of database** | **Sample size (n)** | **No. with findings (%)** | **Sen** | **Spe** |
| Sumathy B et al | 2018 | India | Back propagation neural network | NN | DR | Rajiv Gandhi Eye hospital from Trichy | 59 | 39 (66.1) | 0.95 | 0.90 |
| Fadafen MK et al | 2018 | Iran | Computational model of Human Visual System | Others | DR | DIARETDB1 dataset | 89 | 84 (94.4) | 0.81 | 0.88 |
| Yu S, 2018 et al | 2018 | Australia | Support vector machine | SVM | PDR | MESSIDOR, HRF, DIARETDB0,  Kaggle diabetic retinopathy datasets | 424 | 134 (31.6) | 0.93 | 0.96 |
| Ramachandran N et al | 2018 | New Zealand | Visiona | NN | mtmDR | ODEMS DR screening photos | 485 | 13 (2.7) | 0.85 | 0.80 |
|  |  |  |  |  |  | MESSIDOR dataset | 1200 | 297 (24.8) | 0.96 | 0.90 |
| Malathi K et al | 2018 | India | Recursive Support Vector Machine: Shrinking Edge-Mark model | SVM | DR | Local hospitals | 250 | 201 (80.4) | 1.00 | 0.98 |
| **Study** | **Year** | **Country** | **Algorithms** | **ML category** | **Goal of detection** | **Source of database** | **Sample size (n)** | **No. with findings (%)** | **Sen** | **Spe** |
| Abràmoff MD et al | 2018 | USA | Multilayer convolutional neural networks | NN | mtmDR | Subjects enrolled at 10 sites | 819 | 198 (24.2) | 0.87 | 0.91 |
| Stevenson CH et al | 2019 | New Zealand | Convolutional neural networks | NN | DR | Multiple publicly available fundus datasets | 3170 | 1113 (35.1) | 0.44 | 0.95 |
| Son J et al | 2020 | Korea | Convolutional neural networks | NN | DR | Seoul National University Bundang Hospital Retina Image Archive | 8704 | 158 (1.8) | 0.99 | 0.98 |
|  |  |  |  |  |  | e-Ophtha EX dataset | 82 | 47 (57.3) | 0.89 | 1.0 |
|  |  |  |  |  |  | Indian Diabetic Retinopathy Image Dataset (IDRiD) | 143 | 54 (37.8) | 0.91 | 1.0 |
|  |  |  |  |  |  | MESSIDOR dataset | 1062 | 551 (51.9) | 0.92 | 0.96 |
| Verbraak FD et al | 2019 | Netherlands | IDx-DR-EU-2.1 | NN | mtmDR | Star-SHL, Rotterdam (Netherlands) | 1293 | 62 (4.8) | 0.79 | 0.94 |
|  |  |  |  |  | VTDR |  | 1293 | 17 (1.3) | 1.0 | 0.98 |
| Voets M et al | 2019 | Norway | InceptionV3 model | NN | mtmDR | MESSIDOR-2 dataset. | 1748 | 254 (14.5) | 0.69 | 0.89 |
| **Study** | **Year** | **Country** | **Algorithms** | **ML category** | **Goal of detection** | **Source of database** | **Sample size (n)** | **No. with findings (%)** | **Sen** | **Spe** |
| Pires R et al | 2019 | Brazil | Deep convolutional neural network | NN | mtmDR | EyePACS (Kaggle) training set | 26788 | 5143 (19.2) | 0.88 | 0.92 |
|  |  |  |  |  |  | MESSIDOR-2 dataset | 874 | 128 (14.6) | 0.94 | 0.90 |
|  |  |  | Random forest+ transfer learning | RF |  | DR2 dataset (Department of Ophthalmology, Federal University of São Paulo) | 435 | 98 (22.5) | 0.92 | 0.94 |
| Chowdhury AR et al | 2019 | India | Naïve Bayes | Others | DR | DIARETDB0, DIARETDB1, Tele-ophtha, MESSIDOR, HRF, Retinal image computing and understanding (University of Lincoln), other datasets | 227 | 23 (10.1) | 0.87 | 0.87 |
| Long S et al | 2019 | China | fuzzy C-means clustering + Support vector machine | SVM | DR | DIARETDB1 dataset | 89 | 84 (94.4) | 0.98 | 0.98 |
| Khojasteh P et al | 2019 | Australia | ResNet-50+Support vector machine | Others | DR | DIARETDB1 dataset | 89 | 84 (94.4) | 0.99 | 0.96 |
|  |  |  |  |  |  | e-Ophtha EX dataset | 82 | 47 (57.3) | 0.98 | 0.95 |
| **Study** | **Year** | **Country** | **Algorithms** | **ML category** | **Goal of detection** | **Source of database** | **Sample size (n)** | **No. with findings (%)** | **Sen** | **Spe** |
| Ullah H et al | 2019 | Pakistan | Multi-Layer Feed Forward Perceptron + Chain-like agent genetic algorithm | NN | DR | DIARETDB0, DIARETDB1, DRIVE, Al-Shifa Trust Eye Hospital datasets | 309 | 241 (80.0) | 0.99 | 0.99 |
| Wang H et al | 2020 | China | Deep convolutional neural network +Random forest | Others | DR | e-Ophtha EX dataset | 82 | 47 (57.3) | 0.91 | 0.95 |
|  |  |  |  |  |  | HEI-MED dataset | 169 | 54 (32.0) | 0.91 | 0.91 |
| Xie L et al | 2020 | New Zealand | Diabetic Retinopathy classifier convolutional neural network | NN | DR | EyePACS (Kaggle) dataset | 13305 | 3510 (26.4) | 0.70 | 0.88 |
|  |  |  |  |  | mtmDR |  | 13305 | 600 (4.5) | 0.61 | 0.62 |
| He J et al | 2020 | China | Inception V4 model | NN | DR | PengPu Town Community Hospital | 889 | 143 (16.1) | 0.91 | 0.99 |
|  |  |  |  |  | mtmDR |  | 889 | 101 (11.4) | 0.91 | 0.99 |
| Colomer A et al | 2020 | Spain | Gaussian processes for classification | Others | DR | e-Ophtha EX dataset | 82 | 47 (57.3) | 0.86 | 0.86 |
|  |  |  |  |  |  | DIARETDB1 dataset | 89 | 41 (46.1) | 0.82 | 0.82 |

| **Study** | **Year** | **Country** | **Algorithms** | **ML category** | **Goal of detection** | **Source of database** | **Sample size (n)** | **No. with findings (%)** | **Sen** | **Spe** |
| --- | --- | --- | --- | --- | --- | --- | --- | --- | --- | --- |
| Sosale B et al | 2020 | India | Medios AI algorithm | NN | DR | Outpatient department of Diacon Hospital, Bangalore, India | 900 | 252 (28.0) | 0.83 | 0.96 |
|  |  |  |  |  | mtmDR |  | 900 | 201 (22.3) | 0.93 | 0.93 |
| Shah P et al | 2020 | Singapore | Deep convolutional neural network | NN | DR | Sankara Eye Hospital | 1533 | 1399 (91.3) | 1.00 | 0.99 |
|  |  |  |  |  | mtmDR |  | 1533 | 1378 (89.9) | 0.99 | 0.95 |
|  |  |  |  |  | VTDR |  | 1533 | 899 (58.6) | 0.98 | 0.56 |
|  |  |  |  |  | PDR |  | 1533 | 748 (48.8) | 0.63 | 0.98 |
|  |  |  |  |  | DR | MESSIDOR dataset | 1200 | 654 (54.5) | 0.90 | 0.91 |
|  |  |  |  |  | mtmDR |  | 1200 | 432 (36.0) | 0.95 | 0.97 |
|  |  |  |  |  | VTDR |  | 1200 | 84 (7.0) | 0.92 | 0.93 |
|  |  |  |  |  | PDR |  | 1200 | 24 (2.0) | 0.67 | 1.00 |
| Zago GT et al | 2020 | Brazil | VCG 16 model + Transfer learning | NN | DR | MESSIDOR dataset | 1200 | 654 (54.5) | 0.91 | 0.94 |
|  |  |  |  |  |  | EyePACS (Kaggle) training set | 15919 | 4186 (26.3) | 0.91 | 0.5 |
|  |  |  |  |  |  | IDRiD dataset | 103 | 69 (67.0) | 0.84 | 0.5 |
|  |  |  |  |  |  | DDR dataset | 4105 | 2225 (54.2) | 0.89 | 0.5 |
|  |  |  |  |  |  | DIARETDB0 dataset | 130 | 110 (84.6) | 0.82 | 0.5 |

| **Study** | **Year** | **Country** | **Algorithms** | **ML category** | **Goal of detection** | **Source of database** | **Sample size (n)** | **No. with findings (%)** | **Sen** | **Spe** |
| --- | --- | --- | --- | --- | --- | --- | --- | --- | --- | --- |
| Riaz H et al | 2020 | Korea | DenseNets | NN | DR | MESSIDOR-2 dataset. | 1747 | 730 (41.8) | 0.98 | 0.97 |
|  |  |  |  |  | mtmDR |  | 1747 | 462 (26.4) | 0.99 | 0.99 |
|  |  |  |  |  | VTDR |  | 1747 | 116 (6.6) | 0.99 | 1.00 |
|  |  |  |  |  | PDR |  | 1747 | 38 (2.2) | 0.97 | 1.00 |
|  |  |  |  |  | DR | EyePACS (Kaggle) training set | 17978 | 13824 (76.9) | 0.93 | 0.87 |
|  |  |  |  |  | mtmDR |  | 17978 | 10940 (60.9) | 0.90 | 0.97 |
|  |  |  |  |  | VTDR |  | 17978 | 7575 (42.1) | 0.92 | 0.99 |
|  |  |  |  |  | PDR |  | 17978 | 3794 (21.1) | 0.94 | 1.00 |
| Raumviboonsuk P et al | 2019 | India | Deep learning model | NN | mtmDR | National screening program for DR, Ministry of Public Health of Thailand | 26392 | 3165 (12.0) | 0.97 | 0.95 |
|  |  |  |  |  | VTDR |  | 26392 | 599 (2.3) | 0.94 | 0.98 |
| Li F et al | 2019 | China | Inception-v3 network + Transfer learning | NN | mtmDR | MESSIDOR-2 dataset. | 800 | 264 (33.0) | 0.97 | 0.94 |
| Bhaskaranand M et al | 2019 | USA | EyeArt system v2.0 | NN | mtmDR | EyePACS DR telescreening program (Jan 2014- Sep 2015) | 101710 | 20720 (20.4) | 0.91 | 0.91 |

| **Study** | **Year** | **Country** | **Algorithms** | **ML category** | **Goal of detection** | **Source of database** | **Sample size (n)** | **No. with findings (%)** | **Sen** | **Spe** |
| --- | --- | --- | --- | --- | --- | --- | --- | --- | --- | --- |
| Yang WH et al | 2019 | China | VGGNet + transfer learning | NN | DR | Intelligent Ophthalmology Database of Zhejiang Society for Mathematical Medicine, China. | 500 | 400 (80.0) | 0.99 | 0.88 |
|  |  |  |  |  | mtmDR |  | 500 | 300 (60.0) | 0.98 | 0.97 |
|  |  |  |  |  | VTDR |  | 500 | 200 (40.0) | 0.96 | 0.99 |
| Natarajan S et al | 2019 | India | Medios AI algorithm | NN | DR | Community patients visits (Municipal Corporation of Greater Mumbai, India) | 213 | 26 (12.2) | 0.85 | 0.92 |
|  |  |  |  |  | mtmDR |  | 213 | 15 (7.0) | 1.00 | 0.88 |
| Gulshan V et al | 2019 | India | Deep neural networks | NN | mtmDR | Aravind Eye Hospital | 1905 | 692 (36.3) | 0.89 | 0.92 |
|  |  |  |  |  |  | Sankara Nethralaya | 3747 | 1229 (32.8) | 0.92 | 0.95 |
| Nazir T et al | 2019 | Pakistan | Extreme learning machine | NN | DR | DRIVE dataset | 40 | 7 (17.5) | 0.97 | 0.97 |
|  |  |  |  |  |  | STARE dataset | 400 | 91 (22.8) | 0.94 | 0.97 |
| Bellemo V et al | 2019 | Singapore | Adapted VGGNet architecture + Residual neural network architecture | NN | mtmDR | 5 urban centres in the Copperbelt province of Zambia (Feb - Jun 2012) | 3093 | 706 (22.8) | 0.92 | 0.89 |
|  |  |  |  |  | VTDR |  | 3093 | 171 (5.5) | 0.99 | 0.74 |
| **Study** | **Year** | **Country** | **Algorithms** | **ML category** | **Goal of detection** | **Source of database** | **Sample size (n)** | **No. with findings (%)** | **Sen** | **Spe** |
| Badgujar RD | 2019 | India | Hybrid SMO-GBM classifier | Others | DR | STARE dataset | 40 | 28 (70.0) | 1.00 | 0.92 |
| Hemanth DJ et al | 2018 | India | Modified Hopfield Neural Network | NN | DR | Lotus Eye Care Hospital, India | 270 | 165 (61.1) | 0.99 | 0.99 |
| Kanagasingam Y | 2018 | Australia | Neural network + Transfer-learning | NN | mtmDR | Primary care practice in Midland, Western Australia (Dec 2016- May 2017) | 193 | 2 (1.0) | 1.00 | 0.92 |

^a^Abbreviation: Diabetic retinopathy=DR, Proliferative diabetic retinopathy=PDR, Vision-threatening diabetic retinopathy=VTDR, More-than-mild diabetic retinopathy=mtmDR , NN=Neural network, SVM=Support vector machine, RF=Random forest, No.=Number, Sen=Sensitivity, Spe=Specificity
