# Supplementary material for: Performance and Limitation of Machine Learning Algorithms for Diabetic Retinopathy Screening: Meta-analysis
Source: J Med Internet Res. 2021 Jul 5;23(7):e23863. doi: 10.2196/23863 (PMC8406115; doi:10.2196/23863)

Figure S2. Fagan's plot for diagnosis of different categories of diabetic retinopathy on color fundus photographs

Figure S2a. Any diabetic retinopathy

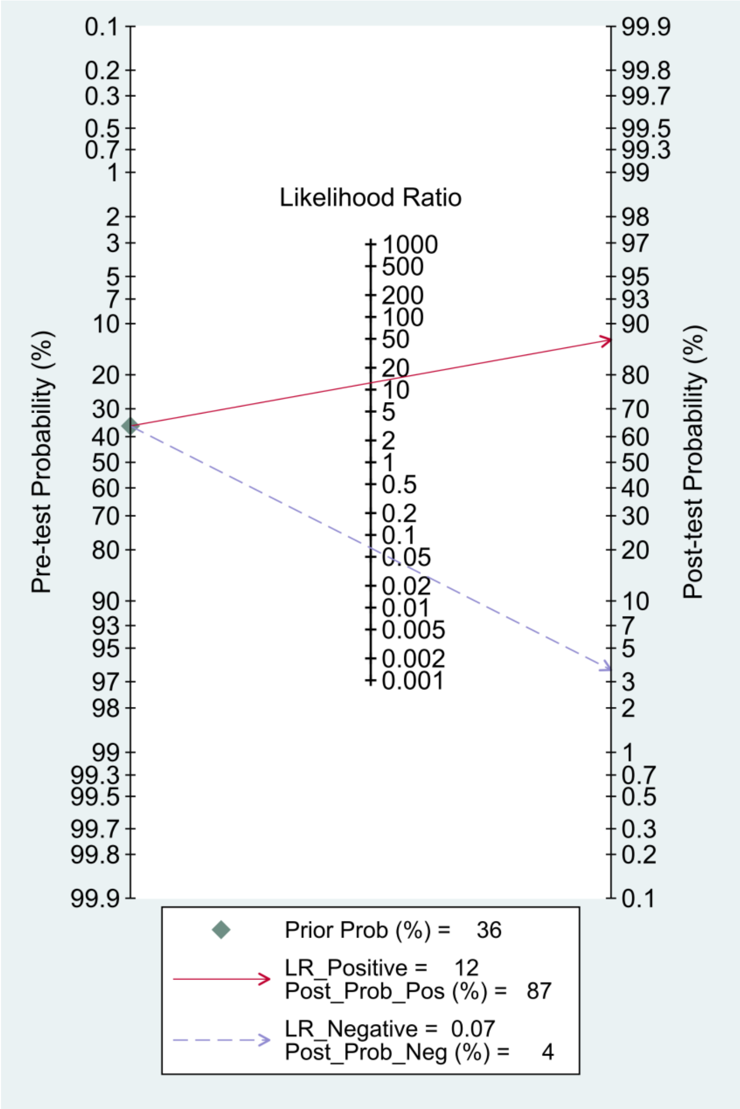

Figure S2b. More-than-mild diabetic retinopathy

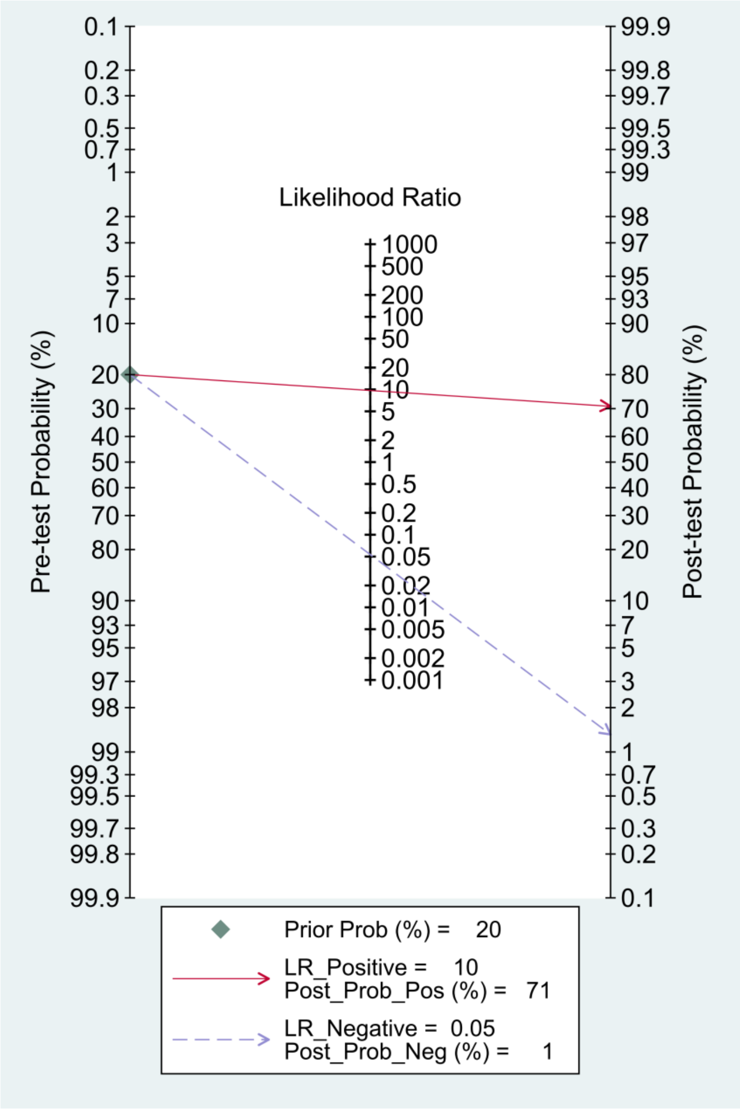

Figure S2c. Vision-threatening diabetic retinopathy

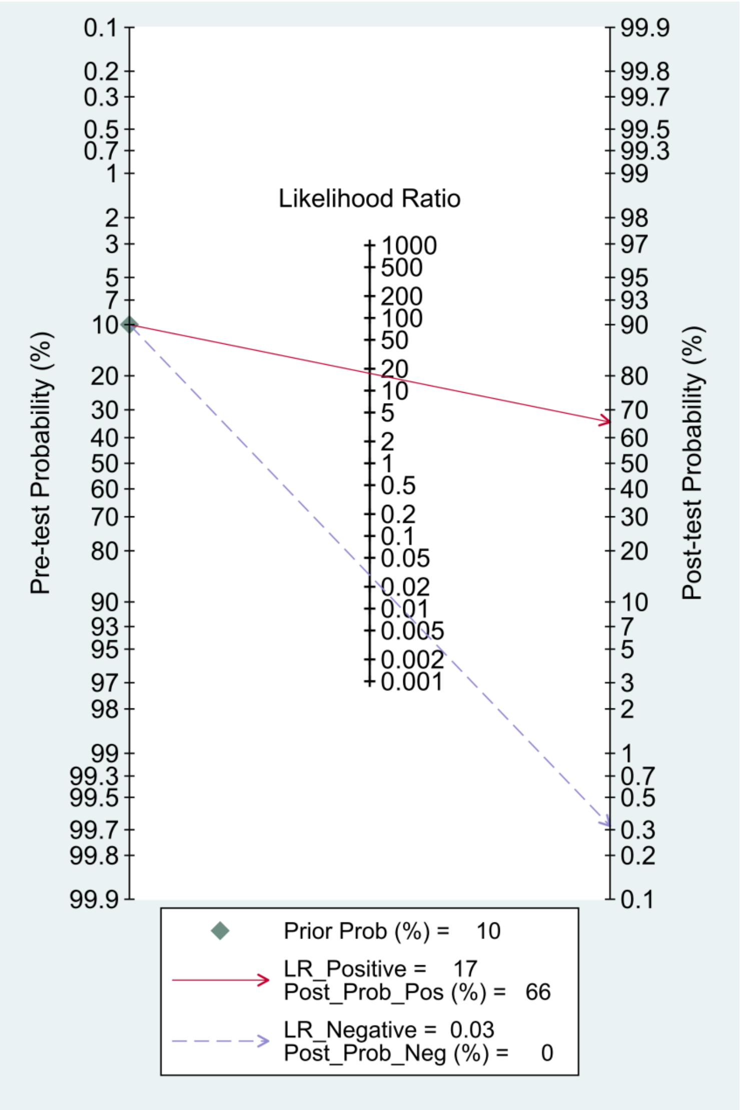

Figure S2d. Proliferative diabetic retinopathy

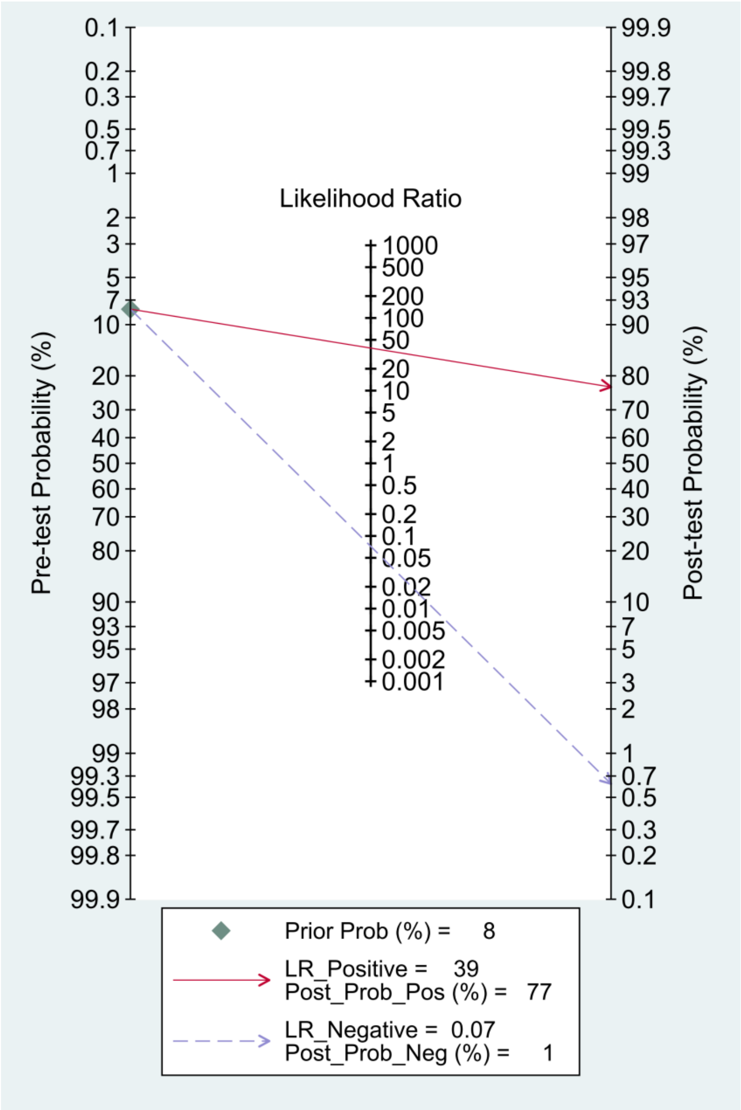

Supplement: Multimedia Appendix 7 [file jmir_v23i7e23863_app7.pdf]
