# Supplementary material for: Performance and Limitation of Machine Learning Algorithms for Diabetic Retinopathy Screening: Meta-analysis
Source: J Med Internet Res. 2021 Jul 5;23(7):e23863. doi: 10.2196/23863 (PMC8406115; doi:10.2196/23863)

Figure S3. Deek's funnel plot for four main types of diabetic retinopathy lesions on color fundus photograph

Figure S3a. Any diabetic retinopathy

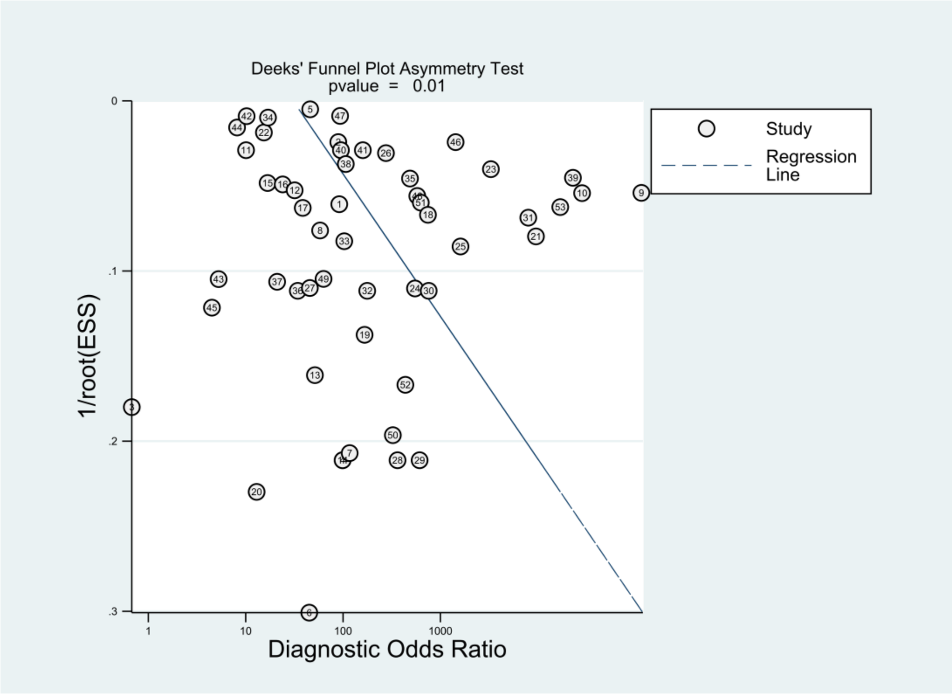

Figure S3b. More-than-mild diabetic retinopathy

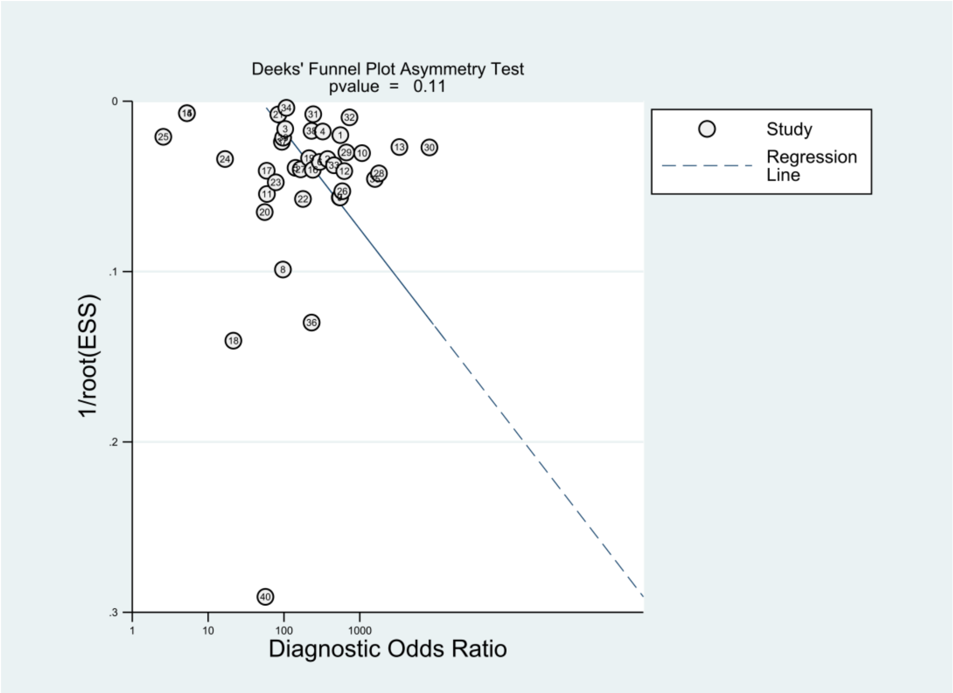

Figure S3c. Vision-threatening diabetic retinopathy

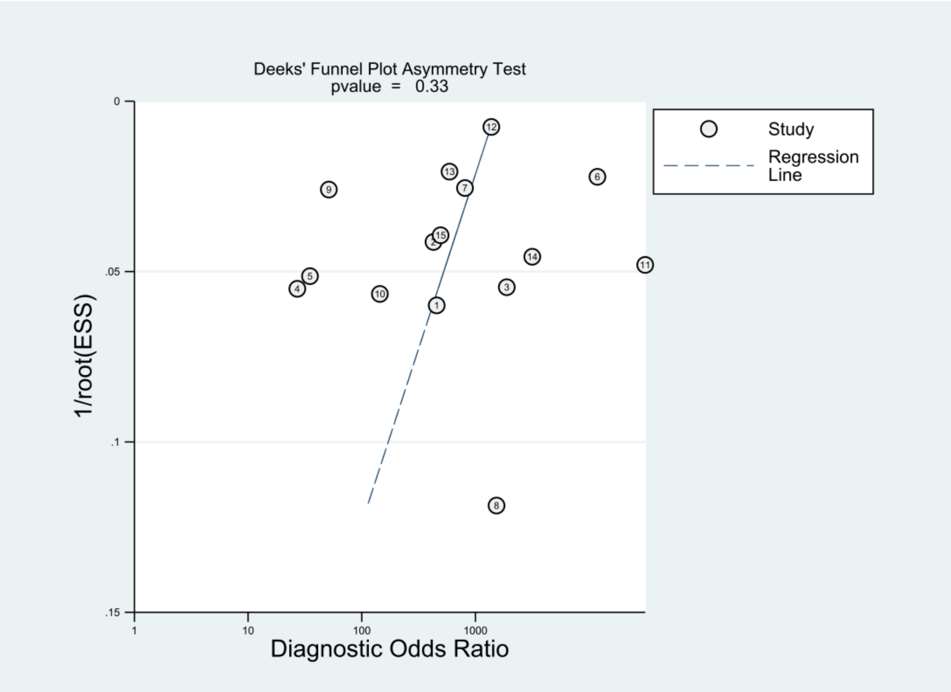

Figure S3d. Proliferative diabetic retinopathy

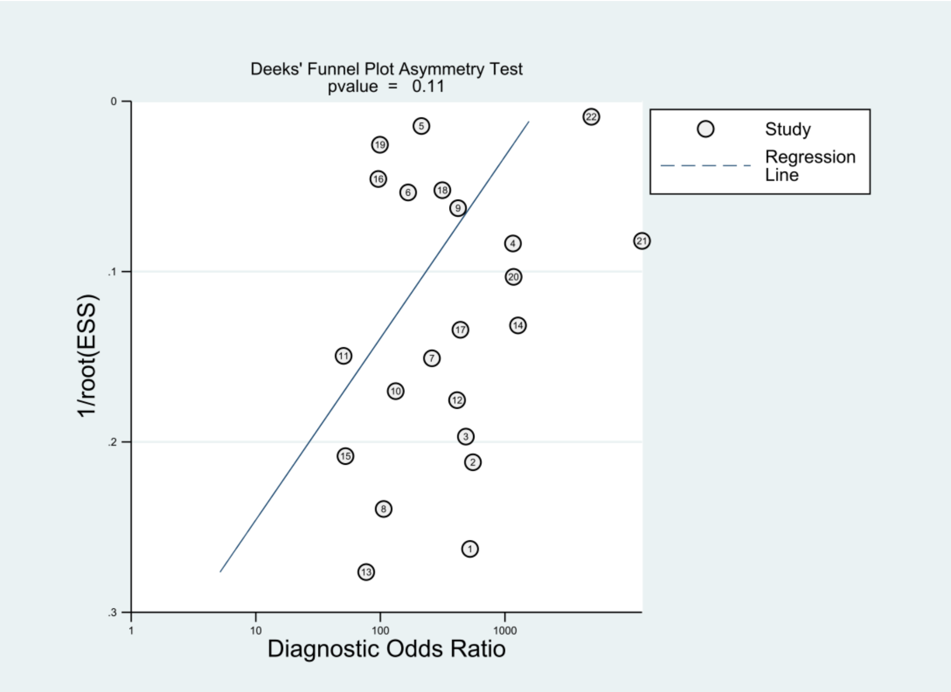

Supplement: Multimedia Appendix 8 [file jmir_v23i7e23863_app8.pdf]
