# Supplementary material for: Performance and Limitation of Machine Learning Algorithms for Diabetic Retinopathy Screening: Meta-analysis
Source: J Med Internet Res. 2021 Jul 5;23(7):e23863. doi: 10.2196/23863 (PMC8406115; doi:10.2196/23863)
Supplement: Multimedia Appendix 9 [file jmir_v23i7e23863_app9.docx]

**
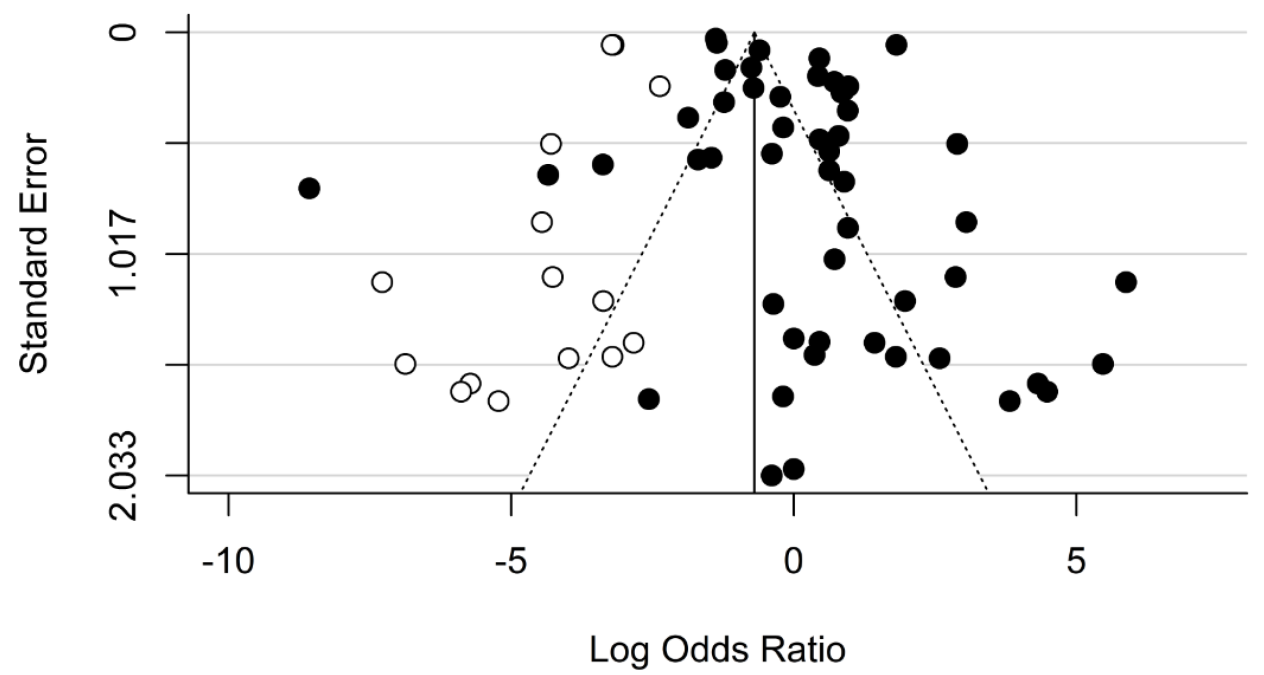
**

^a^The Deek’s test for any diabetic retinopathy of fundoscopic features was significant, indicating the possibility of publication bias. We used trim-and-fill method to incorporate the theoretical missing studies. The original pooled diagnostic OR for any diabetic retinopathy is 1.32 (95% CI: 0.68, 2.53). After incorporating the theoretical missing studies, the update meta-analysis showed a diagnostic OR of 0.50 (95% CI: 0.25, 1.01).
