# Supplementary material for: Performance and Limitation of Machine Learning Algorithms for Diabetic Retinopathy Screening: Meta-analysis
Source: J Med Internet Res. 2021 Jul 5;23(7):e23863. doi: 10.2196/23863 (PMC8406115; doi:10.2196/23863)
Supplement: Multimedia Appendix 10 [file jmir_v23i7e23863_app10.docx]

| **Author** | **Year** | **Country** | **Algorithms** | **ML category** | **Camera** | **FOV** | **Validation database** | **Sen** | **Spe** | **AUC** | **Goal of detection** |
| --- | --- | --- | --- | --- | --- | --- | --- | --- | --- | --- | --- |
| Gulshan V et al | 2016 | USA | Deep learning algorithm | NN | Topcon TRC NW6 | 45° | MESSIDOR-2 dataset | 0.96 | 0.94 | 0.99 | mtmDR |
| Li Z et al | 2018 | China | Convolutional neural network | NN | Canon CR-DGi or Canon CR6-45NM | 45° | National Indigenous Eye Health Survey, Singapore Malay Eye Study, Australian Diabetes Obesity and Lifestyle Study | 0.93 | 0.99 | 0.96 | VTDR |
| Ganesan K et al | 2014 | Singapore | Probabilistic neural network with genetic algorithm | NN | Topcon TRC NW6 | 45° | MESSIDOR dataset | 1.00 | 1.00 | NA | DR |
| Pires R et al | 2015 | Australia | Bag of Visual Words | Others | Canon CR-DGi IOS 30D | 45° | Inala Aboriginal and Torres Strait Islander health care Centre (October 2007 - September 2009) | 1.00 | 0.89 | 0.98 | DR |
| Ting DSW et al | 2017 | Singapore | Deep learning system | NN | Topcon | 45° | Royal Victoria Eye and Ear Hospital | 0.99 | 0.92 | 0.98 | mtmtDR |

^a^Abbreviation: ML=Machine learning, Diabetic retinopathy=DR, Vision-threatening diabetic retinopathy=VTDR, More-than-mild diabetic retinopathy=mtmDR, NN=Neural network, SVM=Support vector machine, RF=Random forest, Sen=Sensitivity, Spe=Specificity, AUC=Area under curve, FOV = Field of view
